# Continence Problems and Mental Health in Adolescents from a UK Cohort

**DOI:** 10.1016/j.eururo.2023.05.013

**Published:** 2023-05-27

**Authors:** Katie Gordon, Naomi Warne, Jon Heron, Alexander von Gontard, Carol Joinson

**Affiliations:** a Population Health Sciences, Bristol Medical School, University of Bristol, Bristol, UK; b Psychiatric Services Graubünden (PDGR), Outpatient Services for Child and Adolescent Psychiatry, Chur, Switzerland; c Governor Kremers Centre, Department of Urology, Maastricht University Medical Centre, Maastricht, The Netherlands; d Centre for Academic Child Health, Population Health Sciences, Bristol Medical School, University of Bristol, Bristol, UK

**Keywords:** Adolescence, Avon Longitudinal Study of Parents and Children, Incontinence, Lower Urinary Tract Symptoms, Mental health, Prospective cohort

## Abstract

**Background:**

Adolescents with continence problems experience unique threats to their psychological well-being, but long-term mental health sequelae are unknown.

**Objective:**

To examine prospective relationships between incontinence/lower urinary tract symptoms (LUTS) and mental health problems in young people.

**Design, setting, and participants:**

A prospective cohort study of young people (*n* = 7332: 3639 males and 3693 females) from a population-based sample was conducted.

**Outcome measurements and statistical analysis:**

We estimated the association between incontinence/LUTS and mental health outcomes using multivariable regression models adjusted for sex, socioeconomic position, developmental level, intelligence quotient, stressful life events, maternal psychopathology, body mass index, and emotional/behavioural problems.

**Results and limitations:**

Daytime wetting and voiding postponement showed the greatest number of associations with mental health problems. All incontinence subtypes/LUTS were associated with increased odds of generalised anxiety disorder (eg, odds ratio for daytime wetting = 3.01, 95% confidence interval [1.78, 5.09], *p* < 0.001) and/or higher anxiety scores. There was also evidence of associations with common mental disorder (eg, voiding postponement: 1.88 [1.46, 2.41], *p* < 0.001), depression (eg, urgency: 1.94 [1.19, 3.14], *p* = 0.008), depressive symptoms (eg, daytime wetting: 1.70 [1.13, 2.56], *p* = 0.01), self-harm thoughts (eg, voiding postponement: 1.52 [1.16, 1.99], *p* = 0.003), and disordered eating (eg, nocturia 1.72 [1.27, 2.34], *p* = 0.001). We are unable to generalise our results to minority ethnic groups, less affluent populations, and non-UK samples.

**Conclusions:**

Young people with incontinence/LUTS are at an increased risk of mental health problems. Further research is needed to establish the direction of causality.

**Patient summary:**

We looked at the association between continence problems and mental health outcomes in young people from a large population-based cohort. Young people with continence problems at the age of 14 yr were more likely to suffer from a range of mental health problems at the age of 18 yr, including common mental disorder, depression, anxiety, self-harm thoughts, and disordered eating. Paediatric continence clinics should address the mental health needs of young people and provide clear and effective care pathways to child and adolescent mental health services.

## 1 Introduction

Incontinence and lower urinary tract symptoms (LUTS) in adolescence are poorly understood due to a lack of empirical evidence. Clinicians are often unaware of the wider issues affecting these adolescents, leading to negative clinical care experience and inadequate treatment [1]. Incontinence is often assumed to resolve during childhood, but persistent, as well as new-onset, cases are not uncommon in adolescence [2,3]. It is estimated that 1–3% of adolescents experience incontinence [3]. Qualitative research with 11–17-yr-olds with incontinence/LUTS found that many young people feel hopeless and pessimistic about their prognosis, and they find it challenging to adhere to treatments [1]. Fears of bullying, social isolation, and “feeling like an outsider”are also common [4]. Young people also report problems in their interpersonal relationships and restrictions to their social life, and feel that they need to conceal their continence problems from friends and romantic partners to appear “normal”[4]. Adolescence is a sensitive period for development of self-concept, and peer rejection can lead to negative self-beliefs, which increase the risk of mental disorder [5]. The secondary school environment is challenging for young people with continence problems, with many experiencing anxiety about restricted access to toilets during class, adverse impacts on learning, and disruptions to lessons and examinations due to frequent toilet trips [4].

Cross-sectional studies have found associations between urinary incontinence in adolescence and adverse psychosocial outcomes including depressive symptoms, peer victimisation, poor self-image, and problems with peer relationships [6]. Many mental health problems first appear in adolescence [7], and young people with incontinence could be at a greater risk of these problems due to the unique myriad of stressors they experience in their daily lives. Despite this, the longer-term mental health sequelae of adolescent continence problems are unknown. Evidence-based knowledge is needed to improve support for this vulnerable group.

We examined whether incontinence (daytime wetting, bedwetting, and soiling) and LUTS (urgency, frequent urination, low voided volume, voiding postponement, and nocturia) at the age of 14 yr are prospectively associated with mental health problems at the age of 18 yr. We studied a range of mental health problems that commonly emerge in adolescence, including depression, anxiety, self-harm, and disordered eating (DE).

## 2 Patients and methods

### 2.1 Participants

We used data from the Avon Longitudinal Study of Parents and Children (ALSPAC). Pregnant women in the former Avon area of South West England with an estimated delivery date between April 1, 1991 and December 31, 1992 were invited to participate. The core sample comprises 14 541 pregnant women with 13 988 children alive at 12 mo. Detailed descriptions of the cohort have been published [8–10]. The study website contains details of all available data (http://www.bristol.ac.uk/alspac/researchers/our-data/). Ethical approval for the study was obtained from the ALSPAC Ethics and Law Committee and the local research ethics committees.

### 2.2 Continence problems at age 14

Participants completed a questionnaire with items on the frequency of incontinence and LUTS during the previous 2 wk, including daytime wetting, bedwetting, and soiling; symptoms of urgency; frequent urination; low voided volume; voiding postponement; and nocturia (Supplementary Table 1).

### 2.3 Mental health problems at age 18

A computerised version of the Clinical Interview Schedule (CIS-R) [11] was used to assess common mental disorder (CMD), International Classification of Diseases (ICD-10) depression, generalised anxiety disorder (GAD), and self-harm (acts and thoughts). Young people completed the CIS-R at a mean age of 17.8 yr (standard deviation = 0.42), hereafter referred to as 18 yr. The Short Mood and Feelings Questionnaire (SMFQ) [12,13] was used to assess depressive symptoms and the Anxiety Sensitivity Index (ASI) [14] assessed physical and mental anxiety symptoms. The Youth Risk Behavior Surveillance System [15] assessed DE behaviours (fasting, purging, binge eating, and excessive exercise). We derived binary variables indicating the presence versus absence of each of these behaviours, a composite variable indicating presence versus absence of any of these behaviours (any DE), and a composite variable for any of the behaviours at the frequency (at least once a week) required for DSM-5 DE diagnosis. Supplementary Table 2 indicates the exact questions and coding for the mental health variables.

### 2.4 Confounders

Analyses were adjusted for sex, socioeconomic position, developmental level and intelligence quotient, maternal stressful life events, maternal depression and anxiety, child’s body mass index, and child’s emotional/behaviour problems (Supplementary Table 3).

### 2.5 Statistical analysis

The primary analysis used multivariable logistic regression, adjusted for the confounders described above, to examine the association between incontinence/LUTS and CMD, ICD-10 depression, high depressive symptoms, GAD symptoms, self-harm acts, and any DE. We conducted secondary analyses of additional aspects of mental health/DE including self-harm thoughts, physical and mental anxiety scores, specific DE behaviours (excessive exercise, fasting, purging, and binge eating), and any DE behaviour occurring one or more times per week (DSM-5 DE). Odds ratios and regression coefficients (*B*) were estimated, as appropriate, with reference to the groups without incontinence/LUTS. We used linear regression for the continuous outcomes (ASI scores). Analyses were performed using Stata version 16 [16].

### 2.6 Missing data

**Fig. 1:**
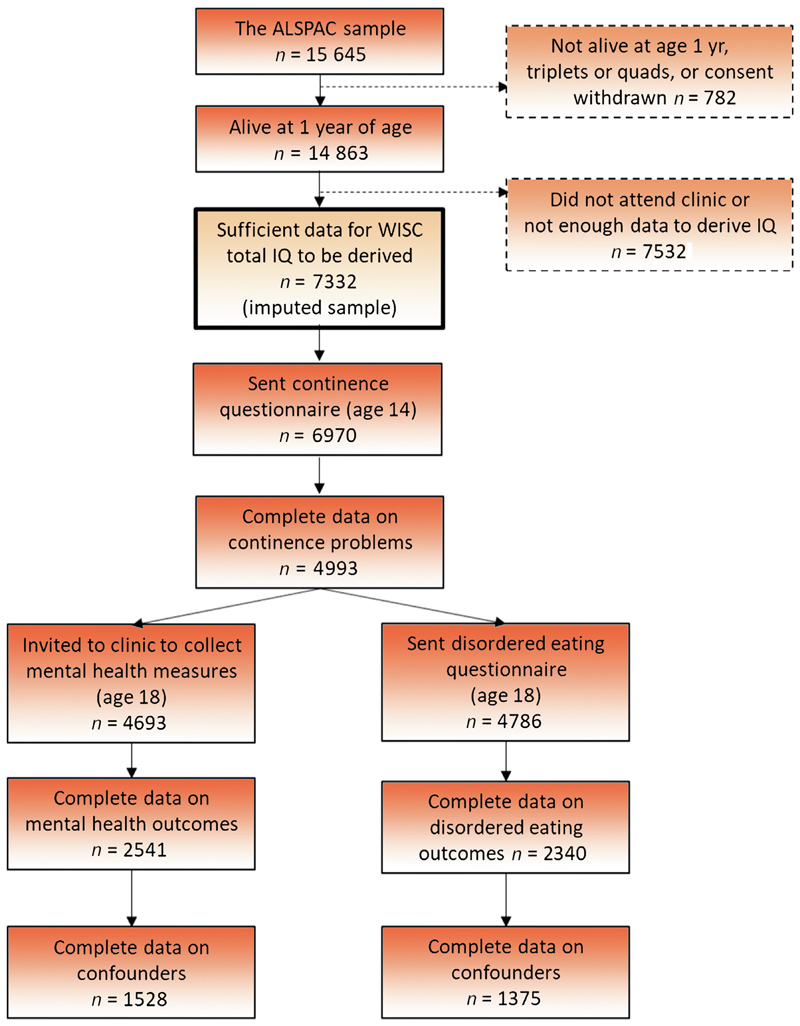
Sample derivation and attrition in ALSPAC. ALSPAC = Avon Longitudinal Study of Parents and Children; IQ = intelligent quotient; WISC = Wechsler Intelligence Scale for Children.

The amount of missing data for each variable is summarised in Supplementary Table 4. The primary analysis focused on an imputed sample of 7332 individuals (3639 males and 3693 females). Full details of the imputation model are provided in the Supplementary material. We also conducted analyses on two complete case samples: 1528 participants who provided all data related to CMD, depression, anxiety, and self-harm, and 1375 participants who provided all DE outcomes (Fig. 1). Supplementary Tables 5 and 6 show the prevalence of participant characteristics by those who did not respond versus those who responded to the 18-yr assessments.

## 3 Results

### 3.1 Descriptive results

Table 1 shows the descriptive statistics for incontinence/LUTS, mental health problems, and confounders in the imputed sample compared with the complete case samples. Descriptive statistics for the secondary outcomes are shown in Supplementary Table 7.

### 3.2 Associations between incontinence/LUTS and mental health outcomes

Table 2 shows the unadjusted and fully adjusted results for the primary analysis (imputed data) examining the associations between incontinence/LUTS and mental health outcomes. Daytime wetting, urgency, and voiding postponement at age 14 were associated with increased odds of mental health outcomes at age 18, including CMD, ICD-10 depression, and high depressive symptoms (daytime wetting and voiding postponement, but not urgency); the strongest associations were found for the presence of GAD symptoms. Most of the associations remained in the fully adjusted models. For example, young people with daytime wetting at age 14 had over a threefold (95% confidence interval: 78–409%) increase in the odds of having at least one GAD symptom at age 18 compared with those without daytime wetting. Bedwetting, frequent urination, and nocturia at age 14 were also associated with GAD symptoms at age 18 in the fully adjusted models. Voiding postponement and nocturia were associated with DE, and there was weak evidence for an association with daytime wetting, bedwetting, and low voided volume. There was little evidence of associations between soiling and mental health outcomes, except for high depressive symptoms. Low voided volume was also associated with high depressive symptoms.

The results for the primary analysis based on the complete data (Supplementary Table 8) showed evidence of strong associations between daytime wetting and CMD, ICD-10 depression, and GAD symptoms. There were some notable differences between the analyses based on the imputed compared with the complete data, suggesting that the complete case analysis was biased by missing data and/or was underpowered. For instance, there was evidence in the imputed analysis, but not the complete case analysis, that voiding postponement is associated with CMD, ICD-10 depression, GAD symptoms, and DE.

Results for the secondary analysis examining additional mental health/DE outcomes are presented in Supplementary Tables 9 (imputed data) and 10 (complete case analysis). There was evidence in the fully adjusted models for associations with higher physical and/or mental anxiety scores (daytime wetting, soiling, urgency, low voided volume, and voiding postponement) and self-harm thoughts (voiding postponement, daytime wetting, and nocturia). There was also evidence for associations with DSM-5 DE (daytime wetting, soiling, and frequent urination, and a weak association with voiding postponement) and DE behaviours including fasting (frequent urination, voiding postponement, and nocturia), purging (daytime wetting), binge eating (daytime wetting, soiling, low voided volume, and nocturia, and a weak association with voiding postponement), and excessive exercise (bedwetting and voiding postponement).

## 4 Discussion

To our knowledge, this is the first prospective cohort study to examine the relationship between incontinence/LUTS and common mental health problems in adolescents. Compared with young people without incontinence/LUTS, adolescents who experienced these problems at age 14 were more likely to have a range of common mental health problems at age 18. Daytime wetting and voiding postponement were associated with the highest number of mental health problems, including CMD, ICD-10 depression, depressive symptoms, and GAD symptoms. Comorbidity has been reported between daytime wetting, voiding postponement, and clinically relevant psychological symptoms in children [17], but studies of adolescents are lacking [3]. We found that all types of incontinence/LUTS were associated with increased odds of GAD symptoms and/or higher anxiety scores. Continence problems in children are associated with higher levels of parent-reported anxiety disorders (including GAD), but no studies have specifically focused on adolescence [18]. We also found associations between incontinence/LUTS and DE behaviours. Inconsistent findings have been reported by studies of small clinical samples that examined whether the prevalence of incontinence is greater in adolescents diagnosed with anorexia than in the general population [19]. Bedwetting was the least prevalent exposure, which might explain why we found fewer (or only weak evidence of) associations with the mental health problems. We also found few associations between soiling and mental health outcomes. Soiling was defined by a positive response to the question of how often do you “dirty your pants during the day”? It is possible that some young people responded positively to this question if they had experienced only slightly soiled underwear (rather than an episode of faecal incontinence), which could have resulted in some nondifferential misclassification of this exposure and could, therefore, have biased the associations with mental health problems towards the null.

### 4.1 Strengths and limitations

Key strengths of this study include the prospective design, availability of self-reported data on a range of incontinence/LUTS types in a large community-based cohort, use of validated self-report questionnaires for mental health problems, and availability of data on a wide range of confounders, including pre-existing emotional/behaviour problems. Further research is needed to determine the direction of association, since it is also possible that mental health problems could cause incontinence/LUTS. Other limitations include the lack of data on treatments for incontinence at age 14 (and possible effects of medications on incontinence), and lack of consideration of constipation as a common contributory cause of continence issues and mental health problems. This is a community-based sample, and therefore, the number of participants who experienced incontinence at high frequencies (eg, wetting/soiling every day) is small compared with clinical samples. We therefore examined the presence versus absence of any incontinence and did not further categorise by frequency because this would have resulted in very small group sizes and lack of precision in our estimates. It is important to note that we found robust associations between incontinence and mental health problems, even when examining incontinence that did not meet the criteria for clinical diagnosis. Another limitation is the possible increase in type 1 errors due to multiple testing. However, we have not based our conclusions purely on *p*-value thresholds (eg, *p* < 0.05) to determine statistical significance, but instead, we consider the effect estimates alongside the strength of evidence indicated by the *p* values and confidence intervals.

An attrition bias due to selective dropout is another potential limitation because the sample with complete data included participants who were more socioeconomically advantaged than the original ALSPAC cohort. Whilst there is evidence that mental health problems are more common in young people from disadvantaged backgrounds [20], the evidence concerning the association between incontinence and socioeconomic background is inconsistent [21]. We used multiple imputation to address a possible bias due to missing data, and compared the results from the analysis of the imputed data and the complete case analysis.

The ALSPAC cohort is predominantly White and affluent [8,9]; hence, we are unable to generalise our results to minority ethnic groups and less affluent populations. Further research in these underserved populations is vital to prevent widening inequalities in health research. Research in non-UK samples is also needed to examine whether these findings generalise to young people from other countries.

### 4.2 Interpretation

The mid-teens are a sensitive period for the development of self-image, and there is evidence that continence problems have an adverse impact on a young person’s psychological well-being [1,4]. Young people with continence problems experience social isolation, perceived stigma, shame, and negative school experiences [4], all of which have been linked to an increased risk of mental health problems [22,23]. All types of incontinence/LUTS were associated with GAD symptoms and/or higher anxiety scores. Daytime wetting and voiding postponement showed the greatest number of associations with mental health problems. There was also strong evidence that urgency was associated with poorer mental health. Urgency is highly unpredictable in nature, and this could contribute to psychological distress. It is notable that daytime wetting was associated with depression/depressive symptoms and GAD symptoms, whilst bedwetting, possibly due to its low prevalence, showed fewer (and weaker) associations with mental health problems. An alternative explanation is that daytime wetting, compared with bedwetting, is difficult to conceal from peers due to the actions required to manage symptoms (eg, frequent toilet trips, changing clothes, and fear of odour from incontinence pads) [4]. Peer acceptance is strongly valued during adolescence, and qualitative research has found that young people with daytime wetting have a strong desire to hide their problems from peers due to shame and fear of being ostracised [4]. The perceived stigma of incontinence and difficulty concealing/controlling symptoms might explain why young people with daytime wetting are at an increased risk of mental health problems.

Voiding postponement in children is associated with social anxiety, behavioural disorders, daytime wetting, and urgency, and is often an acquired and learned behaviour that is used to cope with the perceived embarrassment of needing to use the toilet or to prevent missing out in social situations [24]. In adolescents, voiding postponement has been linked to the fear of using school toilets due to concerns about a lack of privacy, hygiene, or safety (eg, bullying) and is associated with an increased risk of LUTS [25].

The associations between incontinence/LUTS and DE behaviours could be explained by the possibility that incontinence/LUTS and DE could be linked to a need for control and the denial of bodily symptoms [26]. Most cases of incontinence/LUTS in young people are functional, and consequently, clinicians are often unable to give a medical explanation or specific guidance on treatments and prognosis. This can lead to feelings of uncertainty about the controllability of their continence problem, poor adherence to treatments, and pessimism about future treatment success [1]. Illness uncertainty has been linked to maladaptive coping, increased psychological distress, depression, and reduced quality of life [27]. Data were unavailable on DE behaviours that clearly preceded incontinence/LUTS, which could lead to the possibility of reverse causality as an alternative explanation for this association. Consequences of DE (eg, constipation) and behaviours linked to DE (eg, use of laxatives, fluid restriction, and excessive fluid intake) [28] are also associated with incontinence/LUTS.

Incontinence/LUTS were not associated with self-harm acts, but daytime wetting and voiding postponement were associated with self-harm thoughts. Self-harm acts have been associated with behavioural impulsivity, whereas self-harm thoughts are common among those experiencing affective disorders [29]. Clinicians should be aware that young people with daytime wetting and voiding postponement are at an increased risk of self-harm thoughts, given the association with future suicidal behaviour [30].

## 5 Conclusions

Incontinence/LUTS in young people are associated with an increased risk of mental health problems, and adolescents with daytime wetting and voiding postponement are particularly vulnerable. Our findings have important clinical implications in terms of highlighting the need for provision of psychological support for young people with incontinence/LUTS. Clinicians who treat incontinence/LUTS often recognise that young people experience psychological distress and have called for mental health support to be routinely available in paediatric continence clinics. There is a gap between paediatric and adult continence services, and consequently, adolescents with incontinence/LUTS are an underserved population. Transition from paediatric to adult continence services can be poorly managed, and mental health problems are often not assessed or treated, which could exacerbate existing symptoms and affect treatment adherence. Adult urology services need to know that young people transitioning to adult care are at an increased risk of mental health problems. There is also a need for improved support for young people with incontinence/LUTS in secondary schools to manage their symptoms, as well as access to safe, private, and hygienic toilet facilities to prevent young people from avoiding using school toilets. Further research is needed to establish whether the associations we have observed are causal and to examine the possibility of bidirectional causal relationships between mental health problems and incontinence.

## Supplementary Material

Supplementary Materials

## Figures and Tables

**Table 1:** Descriptive information on continence problems, mental health problems, and confounders in the imputed sample compared with the complete case samples

Variable	Imputed sample (*n* = 7332) % or mean (SE)		Mental health sample (*n* = 1528) % or mean (*n* or SD)		Disordered eating sample (*n* = 1375) % or mean (*n* or SD)	
Daytime wetting	3.5%	(0.28)	3.6%	(55)	3.4%	(46)
Bedwetting	3%	(0.26)	2.5%	(39)	2.6%	(36)
Soiling	4.9%	(0.31)	4.5%	(69)	4.9%	(68)
Urgency	5.8%	(0.36)	4.1%	(63)	4.8%	(66)
Frequent urination	3.3%	(0.30)	2.4%	(37)	3.2%	(44)
Low voided volume	4.8%	(0.33)	4.4%	(67)	4.7%	(65)
Voiding postponement	14%	(0.51)	12%	(178)	14%	(184)
Nocturia	9.9%	(0.44)	8.8%	(135)	8.5%	(117)
Common mental disorder	16%	(0.62)	18%	(280)		
ICD-10 depression	8.5%	(0.46)	6.9%	(105)		
High depressive symptoms	22%	(0.69)	13%	(195)		
GAD symptom	6.2%	(0.40)	5.1%	(78)		
Self-harm act	9.5%	(0.49)	8.1%	(123)		
Any disordered eating	32.5%	(0.88)	–	–	33%	(456)
Sex (female)	50%		56%	(853)	59%	(809)
Low parental social class	15%	(0.45)	9.1%	(139)	8.6%	(119)
Ethnicity (non-White)	4.1%	(0.25)	3.1%	(48)	2.9%	(40)
Maternal education						
(O level)	35%	(0.58)	33%	(508)	32%	(443)
Vocational or less	22%	(0.51)	12%	(189)	11%	(154)
Home ownership (rented/other)	9.5%	(0.38)	5.6%	(85)	5.2%	(72)
Family size (3+ children)	4.7%	(0.26)	3.5%	(53)	3.4%	(47)
Material hardship	1.3	(0.03)	1.1	(2.1)	1.1	(2.23)
Maternal stressful life events	3.8	(0.04)	3.8	(2.9)	3.8	(2.8)
Child IQ	104	(0.19)	110	(15)	110	(16)
Child developmental level	0.01	(0.01)	0.02	(0.9)	–0.01	(0.9)
Maternal depressive symptoms	5.7	(0.07)	5.2	(5)	5.2	(5.1)
Maternal anxiety symptoms	4	(0.05)	3.7	(3.3)	3.8	(3.4)
Child behaviour/emotional problems	6.6	(0.06)	5.7	(4.6)	5.6	(4.4)
BMI						
Overweight	27%	(0.59)	24%	(372)	23%	(314)
Underweight	1.9%	(0.19)	2.2%	(34)	1.9%	(26)

BMI = body mass index; GAD = generalised anxiety disorder; ICD-10 = International Classification of Diseases; IQ = intelligence quotient; SD = standard deviation; SE = standard error.

**Table 2:** Odds ratios and 95% confidence intervals for the association between continence problems at age 14 and mental health problems at age 18 (results based on imputed sample, *n* = 7332)

Exposure	Outcome	Unadjusted			Fully adjusted model	
		OR (95% CI)	*p* value		OR (95% CI)	*p* value
Daytime wetting	Common mental disorder	2.38 (1.59, 3.57)	<0.001		1.59 (1.03, 2.47)	0.04
Bedwetting	Common mental disorder	1.77 (1.06, 2.96)	0.03		1.39 (0.78, 2.47)	0.2
Soiling	Common mental disorder	1.56 (1.08, 2.26)	0.02		1.25 (0.84, 1.86)	0.3
Urgency	Common mental disorder	2.07 (1.40, 3.06)	<0.001		1.54 (1.00, 2.36)	0.05
Frequent urination	Common mental disorder	1.83 (1.07, 3.10)	0.03		1.41 (0.80, 2.50)	0.2
Low voided volume	Common mental disorder	1.75 (1.16, 2.65)	0.008		1.45 (0.94, 2.26)	0.09
Voiding postponement	Common mental disorder	2.06 (1.63, 2.60)	<0.001		1.88 (1.46, 2.41)	<0.001
Nocturia	Common mental disorder	1.44 (1.07, 1.94)	0.02		1.19 (0.86, 1.64)	0.3
Daytime wetting	ICD-10 depression	2.48 (1.52, 4.04)	<0.001		1.77 (1.05, 2.98)	0.03
Bedwetting	ICD-10 depression	0.61 (0.22, 1.70)	0.3		0.44 (0.15, 1.31)	0.1
Soiling	ICD-10 depression	1.18 (0.67, 2.05)	0.6		0.99 (0.55, 1.78)	0.9
Urgency	ICD-10 depression	2.43 (1.56, 3.78)	<0.001		1.94 (1.19, 3.14)	0.008
Frequent urination	ICD-10 depression	1.33 (0.67, 2.67)	0.4		1.04 (0.50, 2.14)	0.9
Low voided volume	ICD-10 depression	1.82 (1.10, 2.99)	0.02		1.53 (0.91, 2.57)	0.1
Voiding postponement	ICD-10 depression	1.74 (1.29, 2.35)	<0.001		1.58 (1.15, 2.16)	0.005
Nocturia	ICD-10 depression	1.32 (0.89, 1.96)	0.2		1.11 (0.73, 1.69)	0.6
Daytime wetting	High depressive symptoms	2.26 (1.54, 3.32)	<0.001		1.70 (1.13, 2.56)	0.01
Bedwetting	High depressive symptoms	1.42 (0.88, 2.32)	0.1		1.10 (0.63, 1.89)	0.7
Soiling	High depressive symptoms	1.83 (1.30, 2.59)	0.001		1.52 (1.05, 2.20)	0.03
Urgency	High depressive symptoms	1.27 (0.86, 1.90)	0.2		0.87 (0.56, 1.35)	0.5
Frequent urination	High depressive symptoms	1.35 (0.85, 2.13)	0.2		1.01 (0.62, 1.64)	0.9
Low voided volume	High depressive symptoms	2.21 (1.55, 3.14)	<0.001		1.87 (1.28, 2.73)	0.001
Voiding postponement	High depressive symptoms	1.68 (1.35, 2.10)	<0.001		1.53 (1.21, 1.93)	<0.001
Nocturia	High depressive symptoms	1.55 (1.19, 2.03)	0.001		1.26 (0.95, 1.68)	0.1
Daytime wetting	GAD symptoms	4.11 (2.51, 6.74)	<0.001		3.01 (1.78, 5.09)	<0.001
Bedwetting	GAD symptoms	2.50 (1.22, 5.09)	0.01		2.16 (1.03, 4.52)	0.04
Soiling	GAD symptoms	1.69 (0.96, 2.96)	0.07		1.32 (0.74, 2.35)	0.3
Urgency	GAD symptoms	2.64 (1.54, 4.52)	<0.001		2.09 (1.19, 3.67)	0.01
Frequent urination	GAD symptoms	2.80 (1.48, 5.30)	0.002		2.37 (1.23, 4.57)	0.01
Low voided volume	GAD symptoms	1.22 (0.62, 2.42)	0.6		1.03 (0.51, 2.07)	0.9
Voiding postponement	GAD symptoms	1.74 (1.22, 2.50)	0.003		1.59 (1.10, 2.30)	0.01
Nocturia	GAD symptoms	2.02 (1.31, 3.09)	0.001		1.73 (1.12, 2.68)	0.01
Daytime wetting	Self-harm act	1.66 (0.94, 2.95)	0.08		1.07 (0.58, 1.98)	0.8
Bedwetting	Self-harm act	1.98 (1.11, 3.54)	0.02		1.71 (0.93, 3.16)	0.09
Soiling	Self-harm act	1.36 (0.83, 2.22)	0.2		1.04 (0.62, 1.73)	0.9
Urgency	Self-harm act	0.70 (0.34, 1.45)	0.3		0.49 (0.23, 1.05)	0.06
Frequent urination	Self-harm act	1.63 (0.89, 2.98)	0.1		1.32 (0.70, 2.50)	0.4
Low voided volume	Self-harm act	0.88 (0.50, 1.55)	0.6		0.71 (0.39, 1.27)	0.2
Voiding postponement	Self-harm act	1.17 (0.85, 1.63)	0.3		1.06 (0.75, 1.49)	0.7
Nocturia	Self-harm act	1.35 (0.92, 1.97)	0.1		1.14 (0.77, 1.70)	0.5
Daytime wetting	Any disordered eating	2.06 (1.41, 3.01)	<0.001		1.48 (0.98, 2.25)	0.06
Bedwetting	Any disordered eating	1.51 (0.98, 2.30)	0.06		1.61 (1.00, 2.59)	0.05
Soiling	Any disordered eating	1.68 (1.21, 2.33)	0.002		1.33 (0.92, 1.91)	0.1
Urgency	Any disordered eating	1.33 (0.92, 1.94)	0.1		1.21 (0.79, 1.86)	0.4
Frequent urination	Any disordered eating	1.36 (0.90, 2.07)	0.1		1.24 (0.77, 2.00)	0.4
Low voided volume	Any disordered eating	1.66 (1.11, 2.48)	0.01		1.52 (0.97, 2.40)	0.07
Voiding postponement	Any disordered eating	1.41 (1.13, 1.75)	0.003		1.36 (1.07, 1.74)	0.01
Nocturia	Any disordered eating	1.76 (1.34, 2.31)	<0.001		1.72 (1.27, 2.34)	0.001

BMI = body mass index; CI = confidence interval; GAD = generalised anxiety disorder; ICD-10 = International Classification of Diseases, 10th edition; IQ = intelligence quotient; OR = odds ratio. Fully adjusted models adjusted for child sex, family socioeconomic position, child’s developmental delay and IQ, stressful life events, maternal depression and anxiety, child’s BMI, and earlier emotional/behaviour problems.
