# Supplementary material for: Continence Problems and Mental Health in Adolescents from a UK Cohort
Source: Eur Urol. Author manuscript; Available in PMC 2024 Dec 9. (PMC7617164; doi:10.1016/j.eururo.2023.05.013)
Supplement: Supplementary Materials [file EMS198154-supplement-Supplementary_Materials.zip › 1-s2.0-S030228382302818X-mmc2.docx]

# **Supplementary material**

**Supplementary Table 1. Derivation of the incontinence/LUTS exposure variables from Avon Longitudinal Study of Parents and Children (ALSPAC)**

| **Data collection and age at completion** | **ALSPAC variable** | **Question(s)/measure(s)** | **Responses** | **Final variable coding** |
| --- | --- | --- | --- | --- |
| **Incontinence (**14 years) | | | | |
| ALSPAC ‘travelling, leisure, and school’ questionnaire |  | **How often does the following happen to you?** | |  |
|  | ccp550 | *Daytime wetting*  Wet yourself during the day? | 1: Never  2: Less than once per week  3: Once a week  4: 2-5-times a week  5: Nearly everyday  6: More than once a day  -10, -1: Missing | Recode (1=0) (1/6=1)  0: No  1: Yes |
|  | ccp551 | *Bedwetting*  Wet the bed at night? |  |  |
|  | ccp552 | *Soiling*  Dirty your pants during the day? |  |  |
| **Lower urinary tract symptoms (**14 years) | | | | |
| ALSPAC ‘travelling, leisure, and school’ questionnaire |  | **Over the last two weeks, how often have you:** | |  |
|  | ccp520 | *Urgency*  Had the sudden feeling you need a wee and had to dash to the toilet? | 1: Never  2: A few times  3: Quite often  4: A lot  -10, -1: Missing | Recode (1,2=0) (3/4=1)  0: No  1: Yes |
|  | ccp521 | *Frequent urination*  Had to go to the toilet for a wee more than seven times a day? |  |  |
|  | ccp522 | *Low voided volume*  Passed only a small amount when you went for a wee? |  |  |
|  | ccp524 | *Voiding postponement*  Avoided going for a wee until the last moment because you were concentrating on other activities? |  |  |
|  | ccp527 | *Nocturia*  Woken up to go for a wee? |  |  |

**Supplementary Table 2. Derivation of the mental health outcome variables from Avon Longitudinal Study of Parents and Children (ALSPAC)**

| **Data collection** | **ALSPAC Variable** | **Question(s)/measure(s)** | **Responses** | **Final variable coding** |
| --- | --- | --- | --- | --- |
| **Common mental disorders (18 years)** | | | | |
| Clinical Interview Schedule-Revised^*^ (CIS-R)^*^ | FJCI050 | Total CIS-R score | Continuous variable (range: 0-39) | Recode (<12=0) (≥12=1)  0: No  1: Yes  A score of ≥12 is used to define CMD cases. |
| **Depression (18 years)** | | | | |
| Short Moods and Feelings Questionnaire^*^ | CCXD917 | High depressive symptoms | Continuous variable (range: 0-26)  -10, -1: Missing | Recode (<11=0), (≥11=1)  0: No  1: Yes  Consistent with previous literature, scores of ≥11 were used to define high levels of depressive symptoms, as this threshold has been shown to have high sensitivity and specificity. |
| CIS-R^*^ | FJCI1001 | ICD-10 diagnosis of depression (any severity: mild, moderate, or severe) | 0: Not diagnosed  1: Diagnosed  -10, -4, -1: Missing | 0: No  1: Yes  The depression diagnosis examined in the current study was any depressive episode (mild, moderate or severe). |
| **Anxiety Symptoms (18 years)** | | | | |
| CIS-R^*^ | FJCI602 | Presence of GAD symptoms | Binary variable  0: No  1:Yes  -10, -4, -1: Missing | 0: No  1: Yes |
| Anxiety Sensitivity Index^*^ | FJLE220 | Physical anxiety subscale score | Continuous scale (range: 10-50)  -10, -4, -1: Missing | Continuous variable.  Higher ASI scores indicate greater severity of anxiety |
|  | FJLE221 | Mental anxiety subscale score | Continuous scale (range: 8-40)  -10, -4, -1: Missing |  |
| **Self-Harm (18 years)** | | | | |
| CIS-R^*^  Any acts of self-harm, and any thoughts of self-harm, were recorded as cases, regardless of frequency. | FJCI369 | Have you ever hurt yourself on purpose in any way (e.g., by taking an overdose of pills or by cutting yourself? | 1: No  2: Yes  -10, -4, -1: Missing | If FJCI369 = 2, recode FJCI370 (1=0) (2/5=1):  0: No  1: Yes  If FJCI369 = 1, recode FJCI370 (.=0) |
|  | FJCI370 | How many times have you hurt yourself on purpose in the last year? | 1: None  2: Once  3: 2-5  4: 6-10  5: >10  -10, -4, -1: Missing |  |
|  | FJCI371 | During the past week, have you thought about hurting yourself on purpose? | 1: No  2: Yes, but never commit suicide  3: Yes  -10, -4, -1: Missing | Recode (1=0) (1/2=1)  0: No  1: Yes |
| **Disordered eating behaviours (18 years)** |  |  |  |  |
| Youth Risk Behavior Surveillance System includes items on compensatory behaviours used to control weight, including excessive exercise (that frequently interfered with daily life or resulted in guilt when missing exercise sessions), fasting (not eating for ≥1-day), purging (vomiting, or taking laxatives or other medicines), as well as binge-eating with loss of control. Our primary DE outcome was any disordered eating (any DE), a composite measure covering any instance of these four behaviours. We also examined the specific DE behaviours and an additional composite measure of any of these behaviours occurring ≥1 per week, consistent with DSM-5 diagnostic criteria (DSM-5 DE). |  |  |  |  |
| **Excessive exercise (18 years)** | | | | |
|  |  | **During the past year:** | |  |
|  | cct4105 | How often did you do any exercise (going to the gym, brisk walking, or any other sports activity)? | 1: 5 or more times a week  2: 1-4 times a week  3: 1-3 times a month  4: Less than once a month  5: Never | **Any excessive exercise:**  *Exercise must be accompanied by intent to control weight and/or impairment.*  Recode cct4105 (5=0) (1/4=1)  Recode cct4106 (3=0) (1/2=1)  Recode cct4107 (3=0) (1/2=1)  Recode cct4108 (0=0) (1/3=1)  0: No (if cct41005, cct4106, cct41007 or cct4108 = 0)  1: Yes (if cct41007 = 1 & cct4108 = 1, or if cct41007 =1 & cct4106 = 1)  **DSM-5^†^ excessive exercise:**  Recode cct4105 (3/5=0) (1/2=1)  Recode cct4106 (3=0) (1/2=1)  Recode cct4107 (3=0) (1/2=1), replace = 0 if cct4105 = 0  Recode cct4108 (0/1=0) (2/3=1), replace = 0 if cct4105 = 0  0: (if cct41005, cct4106, cct41007 or cct4108 = 0)  1: Yes (if cct41007 = 1 & cct4108 = 1, or if cct41007 =1 & cct4106 = 1) |
|  | cct4106 | Was it difficult for you to do your work or schoolwork because of the amount of time that you were exercising? | 1: Yes, sometimes  2: Yes, frequently  3: No |  |
|  | cct4107 | Did you exercise in order to lose weight or avoid gaining weight? | 1: Yes, sometimes  2: Yes, frequently  3: No |  |
|  | cct4108 | Did you feel guilty after missing an exercise session? | 0: No  1: Yes, sometimes  2: Yes, frequently  3: Did not miss any exercise sessions |  |
| **Fasting (18 years)** | | | | |
|  | cct4110 | During the past year, how often did you fast (not eat for at least a day) to lose weight or avoid gaining weight? | 1: Never  2: Less than once a month  3: 1-3 times a month  4: Once a week  5: 2 or more times a week | **Any fasting:**  Recode (1=0) (2/5=1)  0: No  1: Yes  **DSM-5^†^ fasting:**  Recode (1/3=0) (4/5=1)  0: No  1: Yes |
| **Purging (18 years)** | | | | |
|  |  | **During the past year:** | |  |
|  | cct4112 | How often did you make yourself throw up (vomit) to lose weight or avoid gaining weight? | 1: Never  2: Less than once a month  3: 1-3 times a month  4: Once a week  5: 2-6 times a week  6: Every day | **Any purging**  Recode cct4112 (1=0) (2/6=1)  Recode cct4115 (3=0) (1/2=1)  Recode cct4116 (1=0) (2/6=1), replace = 0 if cct4115 = 0  0: No (if cct4112 & cct4116=0)  1: Yes (if cct4112 or cct4116=1)  **DSM-5^†^ purging**  Recode cct4112 (1/3=0) (4/6=1)  Recode cct4115 (3=0) (1/2=1)  Recode cct4116 (1/3=0) (4/6=1), replace = 0 if cct4115 = 0  0: No (if cct4112 & cct4116=0)  1: Yes (if cct4112 or cct4116=1) |
|  | cct4115 | Did you take laxatives or other tablets or medicines (diet pills or water tablets) to lose weight or avoid gaining weight? | 1: Yes, laxative  2: Yes, other  3: Never |  |
|  | cct4116 | How often did you take laxatives or other tablets or medicines to lose weight or avoid gaining weight? | 1: Never  2: Less than once a month  3: 1-3 times a month  4: Once a week  5: 2-6 times a week  6: Every day |  |
| **Binge-eating (18 years)**  **ALSPAC Cohort**  n=15,645  **Not alive at 12-months or loss to follow-up**  n=780  **Sent continence questionnaire at age 14**  n=10,687  **Participants who provided data on ≥1 continence exposure**  n=6,882  **Invited to TF4 at age 18**  n=10,071  **Participants who provided data on ≥1 mental health outcome**  n=5,208  **Sent disordered eating questionnaire at age 18**  n=9,953  **Participants who provided data on ≥1 disordered eating outcome**  n=3,383  **Participant alive at 12-months**  n=14,865  **Participants who provided data on both exposure (incontinence) and outcome (mental health) measures**  n=2,358  **Final Sample**  n=1,311  **Excluded due to incomplete confounder^*^ data:**  n=1,047 (insert n for each variable?)  **Figure X. Derivation of final sample from the Avon Longitudinal Study of Parents and Children: participants with self-report data on continence problems at age 14, and common mental health disorders at age 18, and all confounder data.** *Confounders included: sex, body mass index, parental social class, ethnicity, maternal education, home ownership status, maternal hardships, family size, IQ at 8-years, developmental level at 18-months, maternal psychopathology, and earlier incontinence. N.B. Some participants were missing data for more than one confounder variable. *Abbreviations: ALSPAC, Avon Longitudinal Study of Parents and Children; TF4, Teen Focus 4: Clinical Assessment.* | | | | |
|  |  | **During the past year:** | |  |
|  | cct4120 | During the past year, how often did you go on an eating binge? | 1: Less than once a month  2: 1-3 times a month  3: Once a week  4: More than once a week  5: Never | **Any binge-eating**  *Binge-eating must be accompanied by loss of control*.  Recode cct4120 (5=0), (1/4=1)  Recode cct4125 (3=0) (1/2=1)  0: No (if cct4120 or cct4125=0)  1: Yes (if cct4112 & cct4125=1)  **DSM-5^†^ Binge-eating**  Recode cct4120 (5=0) (1/2=0) (3/4=1)  Recode cct4125 (3=0) (1/2=1)  0: No (if cct4120 or cct4125=0)  1: Yes (if cct4112 & cct4125=1) |
|  | cct4125 | Did you feel out of control, like you couldn’t stop eating even if you wanted to stop? | 1: Yes, usually  2: Yes, sometimes  3: No |  |
| **Any disordered eating at any frequency (18 years)** | | | | |
|  |  |  |  | **Presence of any fasting, purging, binge-eating, or excessive exercise:**  0: No  1: Yes |
| **Any disordered eating at DSM-5^†^ frequency (18 years)** | | | | |
|  |  |  |  | **Presence of any DSM-5^†^ fasting, DSM-5^†^ purging, DSM-5^†^ binge-eating, or DSM-5^†^ excessive exercise:**  0: No  1: Yes |
| ^*^Completed at Teen Focus 4 Clinic*.* **^†^**Behaviour occurs ≥1 per week. *Abbreviation: ALSPAC, Avon Longitudinal Study of Parents and Children; DSM-5, Diagnostic and Statistical Manual of Mental Disorders, Fifth Edition.* | | | | |

**Supplementary Table 3. Derivation of the confounder variables from Avon Longitudinal Study of Parents and Children (ALSPAC)**

| **Data collection** | **ALSPAC Variable** | **Question(s)/measure(s)** | **Responses** | **Final variable coding** |
| --- | --- | --- | --- | --- |
| **Child Sex** | | | | |
| Recorded at birth | kz021 | Collected from fieldworkers visiting maternity units at birth | 1: male  2: female  -1: not known | Recode:  0: male  1: female |
| **Parental social class (antenatal questionnaire)** | | | | |
| 1991 British Office of Population and Census Statistics job codes | c755 | Derived variable from questions:   - Actual job, occupation, trade or profession - Please tick which of the following apply to you: foreman, manager, supervisor, leading hand, self-employed, none of these - Type of industry or service given (main things done in job) | 1: I  2: II  3: III (non-manual)  4: III (manual)  5: IV  6: V  65: Armed forces  -1: missing | Recode (1/3=0) (4/6=1) (else=.)  Select highest parental social class i.e., if one parent is non-manual then social class=0:  0: Non-manual: professional, managerial, or skilled professions  1: Manual: partly or unskilled occupations |
|  | c765 |  |  |  |
| **Ethnicity (antenatal questionnaire)** | | | | |
|  | c804 |  | -1: Missing  1: White  2: Non-White | Recode:  0: White  1: Non-White |
| **Maternal education (antenatal questionnaire)** | | | | |
|  | c645a | Derived variable: mother’s highest educational qualification | 1: CSE/none  2: Vocational  3: O-level  4: A-level  5: Degree  -1: Missing | Recode (4/5=0) (3=1) (1/2=2)  0 = A-level or greater  1 = O-level  2 = CSE, vocational or less |
| **Home ownership (when child was aged 10 years)** | | | | |
|  | q2010 | **Is your home:**   1. being bought/mortgaged 2. being bought from council 3. owned - with no mortgage to pay 4. rented from council 5. rented from private landlord - furnished 6. rented from private landlord - unfurnished 7. rented from housing association 8. other (please tick & describe) | 0: Being bought/mortgaged  1: Being bought from council  2: Owned with no mortgage to pay  3: Rented from council  4: Rented from private landlord furnished  5: Rented from private landlord unfurnished  6: Rented from housing association  7: other | Recode (0/2=0) (4/5=0) (3=1) (6/7=1)  0 = Mortgage/owned/privately rented  1 = rent/other |
| **Material hardship (when child was aged 11 years)** | | | | |
| ALSPAC hardship items |  | **How difficult at the moment do you find it to afford these items:** | | Recode r9000-r9004 (5=4)  20–(r9000+r9001+r9002+r9003 +9004) = summed score (range: 0-15)  **Mode imputation:** Replace summed score =. with mode score if at least at least one of r9000-9004 answered  (Higher scores = more material hardship) |
|  | r9000 | a) food | 1: Very difficult  2: Fairly difficult  3: Slightly difficult  4: Not difficult  5: Paid directly by social security |  |
|  | r9001 | b) clothing |  |  |
|  | r9002 | c) heating |  |  |
|  | r9003 | d) rent or mortgage |  |  |
|  | r9004 | e) things you need for your children |  |  |
| **Family size (antenatal questionnaire)** | | | | |
|  | b032 | Derived using parity as proxy. | Continuous variable (range: 0-22) | Recode (0/2=0) (3/22=1)  0 = <3 children  1 = ≥3 children |
| **Developmental delay (when child was aged 18-months)** | | | | |
| Adapted from Denver Developmental Screening Test (*Pediatrics*. 1992;89(1):91–7) | kd680 | Derived variable: Total ALSPAC development score at 18 months (complete case) | Continuous score  -102: Not in correct age range  -101: Missing | Continuous score  (-101, -102 = missing) |
| **Child IQ (when child was aged 8 years)** | | | | |
| Adapted from Wechsler Intelligence Scale for Children (3^rd^ ed) | f8ws112 | Derived variable: Total IQ score at 8-years | Continuous score  -3: Not enough subtests done  -2 Did not start WISC | Continuous score  (-3, -2 = missing) |
| **Stressful life events (when child was aged 11 years)** | | | | |
| ALSPAC ‘life events inventory’ questionnaire |  | **Since the study child’s 9^th^ birthday:** | |  |
|  | r5000 | Respondent's husband/partner died | 0: Relevant text but no box ticked  1: Yes, since study child was 9 or 10  2: Yes, since child’s 11^th^ birthday  3: Yes, both when the study child was 10/11 and since 9^th^ birthday  4: No, did not happen  -10: Not completed  -1: No response | Recode r5000-r5044 (4=0) (0/3=1)  Create summed score (range: 0-45)  **Mode imputation:** Replace summed score =. with mode score if at least at least one of r5000-5044 answered |
|  | r5001 | One of respondent's children has died |  |  |
|  | r5002 | Respondent's friend/relative has died |  |  |
|  | r5003 | One of respondent's children has been ill |  |  |
|  | r5004 | Respondent's husband/partner has been ill |  |  |
|  | r5005 | One of respondent's children has been ill |  |  |
|  | r5006 | Respondent's friend/relative has been ill |  |  |
|  | r5007 | Respondent has been admitted to hospital |  |  |
|  | r5008 | Respondent has been in trouble with the law |  |  |
|  | r5009 | Respondent has been divorced |  |  |
|  | r5010 | Respondent's husband/partner didn't want their child |  |  |
|  | r5011 | Respondent has been very ill |  |  |
|  | r5012 | Respondent's husband/partner lost his job |  |  |
|  | r5013 | Respondent's husband/partner had problems at work |  |  |
|  | r5014 | Respondent had problems at work |  |  |
|  | r5015 | Respondent lost their job |  |  |
|  | r5016 | Respondent's husband/partner went away |  |  |
|  | r5017 | Respondent's husband/partner was in trouble |  |  |
|  | r5018 | Respondent separated from husband/partner |  |  |
|  | r5019 | Respondent's income was reduced |  |  |
|  | r5020 | Respondent argued with their husband/partner |  |  |
|  | r5021 | Respondent argued with their family/friends |  |  |
|  | r5022 | Respondent moved house |  |  |
|  | r5023 | Respondent's husband/partner was physically cruel to them |  |  |
|  | r5024 | Respondent became homeless |  |  |
|  | r5025 | Respondent had a major financial problem |  |  |
|  | r5026 | Respondent got married |  |  |
|  | r5027 | Respondent's husband/partner was physically cruel to their children |  |  |
|  | r5028 | Respondent was physically cruel to their children |  |  |
|  | r5029 | Respondent attempted suicide |  |  |
|  | r5030 | Respondent was convicted of an offence |  |  |
|  | r5031 | Respondent became pregnant |  |  |
|  | r5032 | Respondent started a new job |  |  |
|  | r5033 | Respondent returned to work |  |  |
|  | r5034 | Respondent had a miscarriage |  |  |
|  | r5035 | Respondent had an abortion |  |  |
|  | r5036 | Respondent has taken an examination |  |  |
|  | r5037 | Respondent's husband/partner was emotionally cruel to them |  |  |
|  | r5038 | Respondent's husband/partner has been emotionally cruel to their children |  |  |
|  | r5039 | Respondent has been emotionally cruel to their children |  |  |
|  | r5040 | Respondent's house/car was burgled |  |  |
|  | r5041 | Respondent found a new partner |  |  |
|  | r5042 | One of respondent's child started school |  |  |
|  | r5043 | Respondent's husband/partner started a new job |  |  |
|  | r5044 | Respondent's pet died |  |  |
| **Maternal depression (when child was aged 11 years)** | | | | |
| Edinburgh Postnatal Depression Scale when child was 11-years (*Br J Psychiatry J Ment Sci*. 1987;150:782–6) |  | **In the past seven days:** | |  |
|  | r4010 | I have been able to laugh and see the funny side of things | 1: As much as I always could  2: Not quite so much now  3: Definitely not so much now  4: Not at all  -10, -1: Missing | Recode r4010, r4011, r4013: (1=0) (2=1) (2=3) (4=3)  Recode r4012, r4014-r4019: (4=0), (3=1) (2=2), (1=3)  Sum all 10 items to generate continuous score (range:0-30)  **Mode imputation:** Replace summed score =. with mode score if at least at least one of r4010-r4019 answered |
|  | r4011 | I have looked forward with enjoyment to things | 1: As much as I ever did  2: Rather less than I used to  3: Definitely less that I used to  4: Hardly at all  -10, -1: missing |  |
|  | r4012 | I have blamed myself unnecessarily when things when wrong | 1: Yes, most of the time  2: Yes, some of the time  3: Not very often  4: Never  -10, -1: Missing |  |
|  | r4013 | I have been anxious or worried for no good reason | 1: No, not at all  2: Hardly ever  3: Yes, sometimes  4: Yes, often  -10, -1: Missing |  |
|  | r4014 | I have felt scared or panicky for no good reason | 1: Yes, quite a lot  2: Yes, sometimes  3: No, not much  4: No, not at all  -10, -1: Missing |  |
|  | r4015 | Things have been getting on top of me | 1: Yes, most of the time I haven’t been able to cope  2: Yes, sometimes I haven’t been coping  3: No, most of the time I have coped  4: No, I have been coping as well as ever  -10, -1: Missing |  |
|  | r4016 | I have been so unhappy that I have had difficulty sleeping | 1: Yes, most of the time  2: Yes, sometimes  3: Not very often  4: No, not at all  -10, -1: Missing |  |
|  | r4017 | I have felt sad or miserable | 1: Yes, most of the time  2: Yes, sometimes  3: Not very often  4: No, not at all  -10, -1: Missing |  |
|  | r4018 | I have felt so unhappy that I’ve been crying | 1: Yes, most of the time  2: Yes, quite often  3: Only occasionally  4: Never  -10, -1: Missing |  |
|  | r4019 | The thought of harming myself has occurred to me | 1: Yes, quite often  2: Yes, Sometimes  3: Hardly ever  4: Never  -10, -1: Missing |  |
| **Maternal anxiety (when child was aged 11 years)** | | | | |
| Crown Crisp Experimental Index when child was 11-years (*Br J Med Psychol*. 1988;61(3):255–66) | r4000 | Do you feel upset for no obvious reason? | 1: Very often  2: Often  3: Not very often  4: Never  -10, -1: missing | Recode r4000 r4005 r4006 (3=0) (4=0) (1=2) (2=2)  Recode r4001 r4002 r4004 r4007 (4=0) (3=1) (1=2) (2=2)  Recode r4003 (4=0) (1=2) (2=2) (3=2)  Generate total score: sum of recoded r4000-r4007 (range: 0-24)  **Mode imputation:** Replace summed score =. with mode score if at least at least one of r4007-r4007 answered |
|  | r4001 | Have you felt as though you might faint? |  |  |
|  | r4002 | Do you feel uneasy and restless? |  |  |
|  | r4003 | Do you sometimes feel panicky? |  |  |
|  | r4004 | Do you worry a lot? |  |  |
|  | r4005 | Do you feel strung-up inside? |  |  |
|  | r4006 | Do you ever have the feeling you are going to pieces? |  |  |
|  | r4007 | Do you have bad dreams which upset you when you wake up? |  |  |
|  | ku847 | Wet self during the day |  |  |
|  | ku848 | Wet the bed at night |  |  |
| \| **Child body mass index (when child was aged 13.5 years)** \| \| \| \| \| \| --- \| --- \| --- \| --- \| --- \| \| Clinical measures of height and weight \| fg3134 \| Z-score derived using LMS parameters and 1990 British Growth Reference^1,2^. \| Continuous score  -110, -106, -101: Missing \| Recode z-scores in line with WHO thresholds^3^:  0: Healthy weight (z-score >-2 to <1)  1: Overweight (z-score: ≥1)  2: Underweight (z-score: ≤-2) \|   **Earlier emotional and behavioural problems (when child was aged 11 years 8 months)** | | | | |
| Adapted from Strengths and Difficulties Questionnaire (*Eur Child Adolesc Psychiatry*. 1998;7(3):125–30) | kw660b | Derived variable: SDQ total difficulties score (prorated). | Continuous score (range 0-40)  -10, -6, -5: Missing | Continuous score  Missing = -10, -6, -5 |
| *Abbreviations: ALSPAC, Avon Longitudinal Study of Parents and Children; LMS, lambda-mu-sigma; BMI, body mass index.* | | | | |

**Supplementary Table 4. Amount of missing data for each variable in the substantive models**

| **Variable** | **N missing (/7332)** | **%** |
| --- | --- | --- |
| Daytime wetting | 2188 | 30 |
| Bedtime wetting | 2187 | 30 |
| Soiling | 2195 | 30 |
| Urgency | 2179 | 30 |
| Frequent urination | 2194 | 30 |
| Low voided volume | 2210 | 30 |
| Voiding postponement | 2195 | 30 |
| Nocturia | 2206 | 30 |
| Common Mental Disorder | 3537 | 48 |
| ICD-10 Depression | 3537 | 48 |
| High depressive symptoms (SMFQ) | 3674 | 50 |
| GAD symptoms | 3537 | 48 |
| Physical anxiety score | 3711 | 51 |
| Mental anxiety score | 3593 | 49 |
| Self-harm act | 3537 | 48 |
| Self-harm thoughts | 3537 | 48 |
| Any disordered eating | 4636 | 63 |
| Excessive exercise | 4689 | 64 |
| Fasting | 4645 | 63 |
| Purging | 4648 | 63 |
| Binge-eating | 4650 | 63 |
| DSM-5 frequency disordered eating | 4635 | 63 |
| Sex | 0 | 0 |
| Parental social class | 844 | 11 |
| Ethnicity | 664 | 9 |
| Maternal education | 556 | 7 |
| Home ownership | 1159 | 16 |
| Material hardship | 1514 | 20 |
| Family size/Parity | 550 | 7.5 |
| Developmental level | 2090 | 28 |
| IQ | 0 | 0 |
| Maternal stressful life events | 1459 | 20 |
| Maternal depression | 1463 | 20 |
| Maternal anxiety | 1468 | 20 |
| BMI | 2055 | 28 |
| Earlier behaviour and emotional problems (SDQ) | 1637 | 22 |

**Appendix. Details of the imputation model**

Missing data on exposures, outcomes and confounders were imputed using the multivariate imputation by chained equations approach (mi impute chained command in Stata) under the Missing at Random (MAR) assumption. We restricted the sample to those with complete data on IQ due to a lack of good auxiliary data for IQ, but availability of good auxiliary data for other variables. In addition to variables used in the main analyses, we included auxiliary variables that were likely to be related to the missing data mechanism including incontinence/LUTS (7 and 9 years), depressive symptoms (10 years), depression diagnosis (15 years), emotional problems (7 years), emotional disorder (15 years), anxiety diagnosis (7 years), behaviour/emotional problems (7 and 9 years), self-harm (16 years), suicidal behaviour (11 years), maternal self-harm (during pregnancy), disordered eating (14 and 16 years), and BMI (8, 10, 12.5 years). We also included earlier measures of key indicators of socioeconomic position (material hardship during pregnancy, home ownership and maternal stressful life events at 6 years), maternal mental health (depressive and anxiety symptoms at 2 years) and child developmental level (6 months). We imputed 100 datasets (a decision informed by examining the Monte Carlo errors for the estimated parameters). We repeated analyses across the imputed data sets and combined the estimates using Rubin’s rules^1^.

Reference:

1. White IR, Royston P, Wood AM: Multiple imputation using chained equations: issues and guidance for practice. *Stat Med*. 2011; 30:377–399

**Supplementary Table 5. Prevalence of incontinence/LUTS at age 14 by those who did not versus did respond to the 18-year assessments**

|  | **Participants who *did not* respond to the 18-year assessments of mental health**  **(n= 2,452)** | | **Participants who *did* respond to the 18-year assessments of mental health**  **(n=2,541)** | |  | **Participants who *did* *not* respond to the 18-year assessments of disordered eating (n=2,653)** | | **Participants who *did* respond to the 18-year assessments of disordered eating (n=2,340)** | |  |
| --- | --- | --- | --- | --- | --- | --- | --- | --- | --- | --- |
| **Incontinence/LUTS** | **% or mean**  **(n or SD)** | | **% or mean**  **(n or SD)** | | **p** | **% or mean**  **(n or SD)** | | **% or mean**  **(n or SD)** | | **p** |
| Daytime wetting | 2.5% | (62) | 3.6% | (91) | 0.03 | 2.8% | (75) | 3.3% | (78) | 0.3 |
| Bedwetting | 2.6% | (63) | 2.2% | (55) | 0.3 | 2.1% | (55) | 2.7% | (63) | 0.2 |
| Soiling | 4.5% | (109) | 4.6% | (118) | 0.7 | 4.2% | (111) | 5% | (116) | 0.2 |
| Urgency | 5.3% | (129) | 4.1% | (104) | 0.05 | 5.1% | (134) | 4.2% | (99) | 0.2 |
| Frequent urination | 2.8% | (68) | 2.4% | (60) | 0.4 | 2.3% | (62) | 2.8% | (66) | 0.3 |
| Low voided volume | 4% | (97) | 4.2% | (106) | 0.7 | 4% | (106) | 4% | (97) | 0.8 |
| Voiding postponement | 14% | (355) | 13% | (322) | 0.06 | 14% | (371) | 13% | (306) | 0.4 |
| Nocturia | 9.5% | (234) | 8.3% | (212) | 0.1 | 10% | (266) | 7.7% | (180) | 0.04 |

**Supplementary Table 6. Participant characteristics1 by those who did not versus did respond to the 18-year assessments**

|  | | **Participants who *did not* respond to the 18-year assessments of mental health** | | **Participants who *did* respond to the 18-year assessments of mental health** | |  | **Participants who *did not* respond to the 18-year assessments of disordered eating** | | **Participants who *did* respond to the 18-year assessments of disordered eating** | |  |
| --- | --- | --- | --- | --- | --- | --- | --- | --- | --- | --- | --- |
| **Participant characteristics** | | **% or mean**  **(n or SD)** | | **% or mean**  **(n or SD)** | | **p** | **% or mean**  **(n or SD)** | | **% or mean**  **(n or SD)** | | **p** |
| Sex (female) | | 51% | (1,250) | 58% | (1,481) | <0.001 | 47% | (1,253) | 63% | (1,478) | <0.001 |
| Low parental social class | | 14% | (310) | 9.5% | (222) | <0.001 | 13% | (316) | 10% | (216) | 0.001 |
| Ethnicity (non-white) | | 2.9% | (64) | 3.6% | (85) | 0.18 | 3% | (74) | 3% | (75) | 0.5 |
| Maternal education | |  |  |  |  |  |  |  |  | |  |
|  | (O level) | 36% | (813) | 33% | (791) | <0.001 | 37% | (912) | 31% | (692) | <0.001 |
|  | Vocational or less | 22% | (511) | 14% | (339) |  | 21% | (530) | 14% | (320) |  |
| Home ownership (rented/other) | | 8.3% | (181) | 5.9% | (141) | 0.001 | 7.6% | (180) | 6.4% | (142) | 0.1 |
| Family size (3+ children) | | 4% | (92) | 3.9% | (93) | 0.8 | 4.3% | (105) | 3.6% | (80) | 0.3 |
| Material hardship | | 1.3 | (2.2) | 1.1 | (2.2) | 0.12 | 1.3 | (2.2) | 1.1 | (2.2) | 0.1 |
| Maternal stressful life events | | 3.7 | (2.7) | 3.8 | (2.9) | 0.4 | 3.7 | (2.8) | 3.7 | (2.8) | 0.7 |
| Child IQ | | 103 | (16) | 109 | (15) | <0.001 | 104 | (16) | 109 | (16) | <0.001 |
| Child developmental level | | -0.003 | (0.97) | 0.03 | (0.91) | 0.2 | 0.02 | (0.9) | 0.01 | (0.9) | 0.9 |
| Maternal depressive symptoms | | 5.5 | (5.2) | 5.3 | (5.1) | 0.2 | 5.5 | (5.2) | 5.3 | (5.1) | 0.3 |
| Maternal anxiety symptoms | | 3.9 | (3.6) | 3.8 | (3.4) | 0.4 | 3.9 | (3.5) | 3.9 | (3.5) | 0.9 |
| Child behaviour/emotional problems | | 6.4 | (4.8) | 5.9 | (4.5) | <0.001 | 6.4 | (4.8) | 5.8 | (4.5) | <0.001 |
| BMI | |  |  |  |  |  |  |  |  | |  |
|  | Overweight | 27% | (511) | 26% | (610) | 0.4 | 28% | (601) | 25% | (520) | 0.05 |
|  | Underweight | 1.7% | (31) | 2% | (47) |  | 1.9% | (40) | 1.8% | (38) |  |

1. Total number varies according to availability of data on each characteristic

**Supplementary Table 7. Descriptive information for secondary outcomes (imputed and complete case samples)**

|  | **Imputed sample (n=7,332)** | | **Mental health sample (n=1528)** | | **Disordered eating sample (n=1375)** | |
| --- | --- | --- | --- | --- | --- | --- |
| **Variable** | **% or mean (se)** | | **% or mean (n or SD)** | | **% or mean (n or SD)** | |
| Physical anxiety | 25 | 0.11 | 25 | 7.5 |  |  |
| Mental anxiety | 21 | 0.08 | 22 | 4.8 |  |  |
| Self-harm thoughts | 12% | 0.56 | 9.4% | 144 |  |  |
| Excessive exercise | 22% | 0.81 |  |  | 22% | 310 |
| Fasting | 11% | 0.69 |  |  | 11% | 148 |
| Purging | 6.1% | 0.44 |  |  | 5.5% | 76 |
| Binge-eating | 13% | 0.72 |  |  | 12% | 161 |
| DSM-5 disordered eating | 12% | 0.64 |  |  | 9.6% | 133 |

DSM-5 - Diagnostic and Statistical Manual of Mental Disorders, Fifth Edition

**Supplementary Table 8.** **Associations between incontinence/LUTS and mental health / disordered eating in the complete case data**

**(mental health outcomes n=1,528; disordered eating outcomes n=1,375)**

|  |  | **Unadjusted** | | **Adjusted** | |
| --- | --- | --- | --- | --- | --- |
| **Exposure** | **Outcome** | **OR (95% CI)** | **p** | **OR (95% CI)** | **p** |
| Daytime wetting | Common mental disorder | 2.44 (1.30, 4.56) | 0.005 | 1.95 (1.02, 3.74) | 0.04 |
| Bedwetting | Common mental disorder | 1.01 (0.39, 2.60) | 0.9 | 0.89 (0.33, 2.41) | 0.8 |
| Soiling | Common mental disorder | 1.63 (0.87, 3.04) | 0.1 | 1.31 (0.69, 2.50) | 0.4 |
| Urgency | Common mental disorder | 2.23 (1.22, 4.07) | 0.009 | 2.00 (1.07, 3.73) | 0.03 |
| Frequent urination | Common mental disorder | 1.62 (0.70, 3.73) | 0.3 | 1.44 (0.60, 3.43) | 0.4 |
| Low voided volume | Common mental disorder | 1.21 (0.61, 2.41) | 0.6 | 0.98 (0.48, 1.99) | 0.9 |
| Voiding postponement | Common mental disorder | 1.60 (1.05, 2.42) | 0.03 | 1.45 (0.94, 2.23) | 0.09 |
| Nocturia | Common mental disorder | 1.29 (0.79, 2.11) | 0.3 | 1.26 (0.75, 2.09) | 0.4 |
| **Exposure** | **Outcome** | **OR (95% CI)** | **p** | **OR (95% CI)** | **p** |
| Daytime wetting | ICD-10 depression | 3.22 (1.58, 6.60) | 0.001 | 2.94 (1.40, 6.16)† | 0.004 |
| Bedwetting | ICD-10 depression | 0.35 (0.05, 2.58) | 0.3 | 0.30 (0.04, 2.27) † | 0.2 |
| Soiling | ICD-10 depression | 1.57 (0.70, 3.52) | 0.3 | 1.35 (0.59, 3.10)† | 0.5 |
| Urgency | ICD-10 depression | 3.09 (1.56, 6.11) | 0.001 | 2.90 (1.43, 5.85)† | 0.003 |
| Frequent urination | ICD-10 depression | 1.20 (0.36, 3.98) | 0.7 | 1.13 (0.33, 3.81)† | 0.8 |
| Low voided volume | ICD-10 depression | 1.91 (0.89, 4.10) | 0.1 | 1.63 (0.74, 3.58)† | 0.2 |
| Voiding postponement | ICD-10 depression | 1.40 (0.80, 2.44) | 0.2 | 1.30 (0.74, 2.30)† | 0.4 |
| Nocturia | ICD-10 depression | 1.51 (0.82, 2.77) | 0.2 | 1.52 (0.82, 2.83)† | 0.2 |
| **Exposure** | **Outcome** | **OR (95% CI)** | **p** | **OR (95% CI)** | **p** |
| Daytime wetting | High depressive symptoms | 1.88 (1.03, 3.41) | 0.04 | 1.58 (0.86, 2.93) | 0.1 |
| Bedwetting | High depressive symptoms | 0.97 (0.43, 2.23) | 0.9 | 0.83 (0.35, 1.99) | 0.7 |
| Soiling | High depressive symptoms | 2.18 (1.29, 3.68) | 0.004 | 1.83 (1.06, 3.16) | 0.03 |
| Urgency | High depressive symptoms | 1.05 (0.55, 2.00) | 0.9 | 0.88 (0.45, 1.70) | 0.7 |
| Frequent urination | High depressive symptoms | 1.24 (0.56, 2.73) | 0.6 | 1.07 (0.46, 2.45) | 0.9 |
| Low voided volume | High depressive symptoms | 1.82 (1.05, 3.15) | 0.03 | 1.60 (0.91, 2.83) | 0.1 |
| Voiding postponement | High depressive symptoms | 1.55 (1.07, 2.24) | 0.02 | 1.47 (1.00, 2.16) | 0.05 |
| Nocturia | High depressive symptoms | 1.37 (0.90, 2.09) | 0.1 | 1.31 (0.85, 2.04) | 0.2 |
| **Exposure** | **Outcome** | **OR (95% CI)** | **p** | **OR (95% CI)** | **p** |
| Daytime wetting | GAD symptoms | 3.41 (1.55, 7.50) | 0.002 | 2.80 (1.24, 6.32)† | 0.013 |
| Bedwetting | GAD symptoms | 2.19 (0.76, 6.31) | 0.1 | 2.04 (0.68, 6.11)† | 0.203 |
| Soiling | GAD symptoms | 1.48 (0.58, 3.80) | 0.4 | 1.25 (0.48, 3.26)† | 0.6 |
| Urgency | GAD symptoms | 2.45 (1.08, 5.58) | 0.03 | 2.20 (0.95, 5.13)† | 0.07 |
| Frequent urination | GAD symptoms | 3.81 (1.54, 9.44) | 0.004 | 3.44 (1.34, 8.81)† | 0.01 |
| Low voided volume | GAD symptoms | 1.53 (0.60, 3.93) | 0.4 | 1.27 (0.49, 3.31)† | 0.6 |
| Voiding postponement | GAD symptoms | 1.12 (0.57, 2.22) | 0.7 | 1.00 (0.50, 2.00)† | 0.9 |
| Nocturia | GAD symptoms | 1.56 (0.78, 3.10) | 0.2 | 1.51 (0.75, 3.06)† | 0.2 |
| **Exposure** | **Outcome** | **OR (95% CI)** | **p** | **OR (95% CI)** | **p** |
| Daytime wetting | Self-harm act | 1.42 (0.60, 3.38) | 0.4 | 1.22 (0.50, 2.98)† | 0.6 |
| Bedwetting | Self-harm act | 2.59 (1.12, 5.99) | 0.03 | 2.77 (1.15, 6.67)† | 0.02 |
| Soiling | Self-harm act | 1.53 (0.72, 3.28) | 0.3 | 1.32 (0.61, 2.90)† | 0.5 |
| Urgency | Self-harm act | 0.56 (0.17, 1.81) | 0.3 | 0.49 (0.15, 1.61)† | 0.2 |
| Frequent urination | Self-harm act | 1.01 (0.31, 3.33) | 0.9 | 0.88 (0.26, 3.01)† | 0.8 |
| Low voided volume | Self-harm act | 0.92 (0.36, 2.33) | 0.8 | 0.76 (0.30, 1.97)† | 0.6 |
| Voiding postponement | Self-harm act | 1.15 (0.66, 1.99) | 0.6 | 0.99 (0.56, 1.74)† | 0.9 |
| Nocturia | Self-harm act | 1.24 (0.68, 2.27) | 0.5 | 1.20 (0.65, 2.23)† | 0.6 |
| **Exposure** | **Outcome** | **OR (95% CI)** | **p** | **OR (95% CI)** | **p** |
| Daytime wetting | Any disordered eating | 2.07 (1.15, 3.73) | 0.02 | 1.58 (0.84, 2.99) | 0.2 |
| Bedwetting | Any disordered eating | 1.45 (0.74, 2.85) | 0.3 | 1.41 (0.67, 2.98) | 0.4 |
| Soiling | Any disordered eating | 1.35 (0.82, 2.22) | 0.2 | 1.12 (0.66, 1.92) | 0.7 |
| Urgency | Any disordered eating | 1.24 (0.75, 2.07) | 0.4 | 1.22 (0.70, 2.11) | 0.5 |
| Frequent urination | Any disordered eating | 1.16 (0.62, 2.16) | 0.6 | 1.08 (0.55, 2.12) | 0.8 |
| Low voided volume | Any disordered eating | 1.78 (1.08, 2.94) | 0.02 | 1.65 (0.96, 2.85) | 0.07 |
| Voiding postponement | Any disordered eating | 1.35 (0.98, 1.86) | 0.06 | 1.28 (0.90, 1.81) | 0.2 |
| Nocturia | Any disordered eating | 1.51 (1.02, 2.22) | 0.04 | 1.58 (1.04, 2.41) | 0.03 |

GAD – Generalised Anxiety Disorder; ICD-10 - International Classification of Diseases, Tenth Revision. †Analyses performed on n=1494 due to 34 observations being dropped (underweight BMI predicted outcome perfectly).

The adjusted model includes the following confounders: sex, socioeconomic indicators, child IQ, developmental level, maternal stressful life events, maternal depression, maternal anxiety, child Body Mass Index and earlier emotional/behaviour problems.

**Supplementary Table 9. Associations between incontinence/LUTS and additional mental health / disordered eating outcomes in the imputed data (n=7,332)**

|  |  | **Unadjusted** | | **Adjusted** | |
| --- | --- | --- | --- | --- | --- |
| **Exposure** | **Outcome** | **B (95% CI)** | **p** | **B (95% CI)** | **p** |
| Daytime wetting | Physical anxiety | 2.15 (0.77, 3.52) | 0.002 | 0.92 (-0.42, 2.25) | 0.2 |
| Bedwetting | Physical anxiety | 0.19 (-1.51, 1.89) | 0.8 | 0.16 (-1.48, 1.81) | 0.8 |
| Soiling | Physical anxiety | 2.26 (1.04, 3.47) | <0.001 | 1.34 (0.15, 2.52) | 0.03 |
| Urgency | Physical anxiety | 1.93 (0.69, 3.16) | 0.002 | 1.36 (0.12, 2.60) | 0.03 |
| Frequent urination | Physical anxiety | 1.10 (-0.51, 2.70) | 0.2 | 0.60 (-0.94, 2.15) | 0.4 |
| Low voided volume | Physical anxiety | 1.94 (0.56, 3.31) | 0.006 | 1.43 (0.08, 2.79) | 0.04 |
| Voiding postponement | Physical anxiety | 1.89 (1.17, 2.61) | <0.001 | 1.64 (0.95, 2.33) | <0.001 |
| Nocturia | Physical anxiety | 1.10 (0.20, 2.00) | 0.017 | 0.81 (-0.07, 1.69) | 0.07 |
| **Exposure** | **Outcome** | **B (95% CI)** | **p** | **B (95% CI)** | **p** |
| Daytime wetting | Mental anxiety | 1.58 (0.65, 2.51) | 0.001 | 1.08 (0.15, 2.02) | 0.02 |
| Bedwetting | Mental anxiety | 0.65 (-0.52, 1.83) | 0.3 | 0.37 (-0.80, 1.55) | 0.5 |
| Soiling | Mental anxiety | 1.43 (0.59, 2.28) | 0.001 | 1.17 (0.32, 2.02) | 0.007 |
| Urgency | Mental anxiety | 1.00 (0.16, 1.83) | 0.02 | 0.67 (-0.16, 1.51) | 0.1 |
| Frequent urination | Mental anxiety | 0.55 (-0.54, 1.64) | 0.3 | 0.29 (-0.77, 1.34) | 0.6 |
| Low voided volume | Mental anxiety | 1.60 (0.66, 2.54) | 0.001 | 1.36 (0.41, 2.31) | 0.005 |
| Voiding postponement | Mental anxiety | 1.45 (0.99, 1.92) | <0.001 | 1.31 (0.85, 1.78) | <0.001 |
| Nocturia | Mental anxiety | 0.52 (-0.18, 1.22) | 0.1 | 0.34 (-0.35, 1.03) | 0.3 |
| **Exposure** | **Outcome** | **OR (95% CI)** | **p** | **OR (95% CI)** | **p** |
| Daytime wetting | Self-harm thoughts | 2.48 (1.58, 3.91) | <0.001 | 1.83 (1.12, 2.98) | 0.01 |
| Bedwetting | Self-harm thoughts | 1.36 (0.70, 2.66) | 0.4 | 1.14 (0.56, 2.31) | 0.7 |
| Soiling | Self-harm thoughts | 1.70 (1.11, 2.61) | 0.01 | 1.37 (0.88, 2.14) | 0.2 |
| Urgency | Self-harm thoughts | 1.19 (0.70, 2.03) | 0.5 | 0.88 (0.51, 1.54) | 0.7 |
| Frequent urination | Self-harm thoughts | 1.05 (0.55, 2.01) | 0.9 | 0.82 (0.42, 1.62) | 0.6 |
| Low voided volume | Self-harm thoughts | 1.67 (1.11, 2.52) | 0.01 | 1.42 (0.92, 2.18) | 0.1 |
| Voiding postponement | Self-harm thoughts | 1.64 (1.27, 2.12) | <0.001 | 1.52 (1.16, 1.99) | 0.003 |
| Nocturia | Self-harm thoughts | 1.65 (1.19, 2.28) | 0.003 | 1.40 (1.00, 1.96) | 0.05 |
| **Exposure** | **Outcome** | **OR (95% CI)** | **p** | **OR (95% CI)** | **p** |
| Daytime wetting | Fasting | 1.77 (1.05, 2.97) | 0.03 | 1.12 (0.65, 1.94) | 0.690 |
| Bedwetting | Fasting | 1.63 (0.87, 3.03) | 0.1 | 1.43 (0.71, 2.87) | 0.3 |
| Soiling | Fasting | 1.77 (1.17, 2.67) | 0.007 | 1.32 (0.84, 2.08) | 0.2 |
| Urgency | Fasting | 1.83 (1.16, 2.88) | 0.009 | 1.41 (0.84, 2.34) | 0.2 |
| Frequent urination | Fasting | 2.84 (1.66, 4.85) | <0.001 | 2.43 (1.32, 4.46) | 0.004 |
| Low voided volume | Fasting | 1.67 (1.04, 2.68) | 0.03 | 1.39 (0.82, 2.37) | 0.2 |
| Voiding postponement | Fasting | 1.80 (1.33, 2.44) | <0.001 | 1.69 (1.21, 2.35) | 0.002 |
| Nocturia | Fasting | 1.99 (1.39, 2.83) | <0.001 | 1.69 (1.15, 2.48) | 0.008 |
| **Exposure** | **Outcome** | **OR (95% CI)** | **p** | **OR (95% CI)** | **p** |
| Daytime wetting | Purging | 2.77 (1.59, 4.85) | <0.001 | 1.88 (1.02, 3.46) | 0.04 |
| Bedwetting | Purging | 1.82 (0.86, 3.84) | 0.1 | 1.78 (0.77, 4.11) | 0.2 |
| Soiling | Purging | 2.07 (1.22, 3.49) | 0.007 | 1.55 (0.89, 2.72) | 0.1 |
| Urgency | Purging | 1.68 (0.87, 3.21) | 0.1 | 1.37 (0.68, 2.76) | 0.4 |
| Frequent urination | Purging | 1.07 (0.44, 2.60) | 0.9 | 0.86 (0.34, 2.16) | 0.7 |
| Low voided volume | Purging | 1.88 (1.05, 3.36) | 0.03 | 1.69 (0.91, 3.13) | 0.09 |
| Voiding postponement | Purging | 1.53 (0.99, 2.35) | 0.05 | 1.39 (0.88, 2.19) | 0.1 |
| Nocturia | Purging | 1.72 (1.06, 2.79) | 0.03 | 1.55 (0.92, 2.60) | 0.1 |
| **Exposure** | **Outcome** | **OR (95% CI)** | **p** | **OR (95% CI)** | **p** |
| Daytime wetting | Binge-eating | 3.24 (2.13, 4.92) | <0.001 | 2.26 (1.42, 3.59) | 0.001 |
| Bedwetting | Binge-eating | 1.29 (0.73, 2.28) | 0.4 | 1.15 (0.61, 2.14) | 0.7 |
| Soiling | Binge-eating | 2.06 (1.35, 3.14) | 0.001 | 1.58 (1.02, 2.46) | 0.04 |
| Urgency | Binge-eating | 1.69 (1.05, 2.73) | 0.03 | 1.36 (0.81, 2.28) | 0.2 |
| Frequent urination | Binge-eating | 1.18 (0.66, 2.12) | 0.6 | 0.94 (0.50, 1.75) | 0.8 |
| Low voided volume | Binge-eating | 2.04 (1.31, 3.18) | 0.002 | 1.79 (1.12, 2.85) | 0.01 |
| Voiding postponement | Binge-eating | 1.43 (1.06, 1.94) | 0.02 | 1.33 (0.97, 1.83) | 0.08 |
| Nocturia | Binge-eating | 1.65 (1.18, 2.31) | 0.004 | 1.45 (1.01, 2.07) | 0.04 |
| **Exposure** | **Outcome** | **OR (95% CI)** | **p** | **OR (95% CI)** | **p** |
| Daytime wetting | Excessive exercise | 1.77 (1.14, 2.76) | 0.01 | 1.40 (0.86, 2.27) | 0.2 |
| Bedwetting | Excessive exercise | 1.43 (0.88, 2.32) | 0.1 | 1.76 (1.01, 3.05) | 0.04 |
| Soiling | Excessive exercise | 1.37 (0.95, 1.97) | 0.09 | 1.11 (0.76, 1.64) | 0.6 |
| Urgency | Excessive exercise | 0.87 (0.57, 1.33) | 0.5 | 0.87 (0.55, 1.37) | 0.5 |
| Frequent urination | Excessive exercise | 1.21 (0.74, 1.99) | 0.4 | 1.21 (0.70, 2.11) | 0.5 |
| Low voided volume | Excessive exercise | 1.63 (1.08, 2.45) | 0.02 | 1.56 (0.98, 2.46) | 0.06 |
| Voiding postponement | Excessive exercise | 1.35 (1.05, 1.75) | 0.02 | 1.36 (1.03, 1.80) | 0.03 |
| Nocturia | Excessive exercise | 1.56 (1.16, 2.11) | 0.004 | 1.64 (1.18, 2.28) | 0.003 |
| **Exposure** | **Outcome** | **OR (95% CI)** | **p** | **OR (95% CI)** | **p** |
| Daytime wetting | DSM-5 DE | 2.35 (1.42, 3.89) | 0.001 | 1.83 (1.04, 3.20) | 0.04 |
| Bedwetting | DSM-5 DE | 1.39 (0.72, 2.70) | 0.3 | 1.25 (0.60, 2.61) | 0.5 |
| Soiling | DSM-5 DE | 2.13 (1.37, 3.32) | 0.001 | 1.72 (1.07, 2.78) | 0.03 |
| Urgency | DSM-5 DE | 1.44 (0.87, 2.37) | 0.1 | 1.09 (0.64, 1.88) | 0.7 |
| Frequent urination | DSM-5 DE | 2.26 (1.27, 4.01) | 0.006 | 1.85 (0.99, 3.47) | 0.05 |
| Low voided volume | DSM-5 DE | 1.85 (1.11, 3.08) | 0.02 | 1.59 (0.91, 2.77) | 0.09 |
| Voiding postponement | DSM-5 DE | 1.52 (1.08, 2.14) | 0.01 | 1.40 (0.98, 2.01) | 0.06 |
| Nocturia | DSM-5 DE | 1.56 (1.06, 2.32) | 0.03 | 1.28 (0.83, 1.97) | 0.3 |

DSM-5 DE- Disordered eating at Diagnostic and Statistical Manual of Mental Disorders, Fifth Edition frequency (at least once a week).

The adjusted model includes the following confounders: sex, socioeconomic indicators, child IQ, developmental level, maternal stressful life events, maternal depression, maternal anxiety, child Body Mass Index and earlier emotional/behaviour problems.

**Supplementary Table 10. Associations between incontinence/LUTS and additional mental health / disordered eating secondary outcomes in the complete case data (mental health outcomes n=1,528; disordered eating outcomes n=1,375)**

|  |  | **Unadjusted** | | **Adjusted** | |
| --- | --- | --- | --- | --- | --- |
| **Exposure** | **Outcome** | **B (95% CI)** | **p** | **B (95% CI)** | **p** |
| Daytime wetting | Physical anxiety | 1.87 (-0.16, 3.89) | 0.07 | 1.12 (-0.87, 3.10) | 0.3 |
| Bedwetting | Physical anxiety | -1.28 (-3.67, 1.11) | 0.3 | -1.23 (-3.58, 1.11) | 0.3 |
| Soiling | Physical anxiety | 1.13 (-0.69, 2.94) | 0.2 | 0.35 (-1.44, 2.13) | 0.7 |
| Urgency | Physical anxiety | 2.50 (0.60, 4.39) | 0.01 | 2.11 (0.25, 3.97) | 0.03 |
| Frequent urination | Physical anxiety | 0.31 (-2.14, 2.76) | 0.8 | 0.01 (-2.39, 2.41) | 0.9 |
| Low voided volume | Physical anxiety | 1.38 (-0.47, 3.22) | 0.1 | 0.93 (-0.87, 2.74) | 0.3 |
| Voiding postponement | Physical anxiety | 1.65 (0.48, 2.82) | 0.006 | 1.52 (0.37, 2.67) | 0.01 |
| Nocturia | Physical anxiety | 1.04 (-0.29, 2.37) | 0.1 | 1.03 (-0.27, 2.33) | 0.1 |
| **Exposure** | **Outcome** | **B (95% CI)** | **p** | **B (95% CI)** | **p** |
| Daytime wetting | Mental anxiety | 0.97 (-0.32, 2.27) | 0.1 | 0.80 (-0.50, 2.10) | 0.2 |
| Bedwetting | Mental anxiety | -0.70 (-2.23, 0.83) | 0.4 | -0.94 (-2.47, 0.59) | 0.2 |
| Soiling | Mental anxiety | 1.09 (-0.07, 2.25) | 0.06 | 0.96 (-0.20, 2.13) | 0.1 |
| Urgency | Mental anxiety | 1.39 (0.18, 2.60) | 0.02 | 1.29 (0.08, 2.51) | 0.04 |
| Frequent urination | Mental anxiety | 0.33 (-1.24, 1.90) | 0.7 | 0.29 (-1.28, 1.85) | 0.7 |
| Low voided volume | Mental anxiety | 1.29 (0.11, 2.46) | 0.03 | 1.11 (-0.07, 2.29) | 0.07 |
| Voiding postponement | Mental anxiety | 1.12 (0.37, 1.87) | 0.003 | 1.02 (0.27, 1.78) | 0.008 |
| Nocturia | Mental anxiety | 0.46 (-0.39, 1.31) | 0.3 | 0.42 (-0.43, 1.27) | 0.3 |
| **Exposure** | **Outcome** | **OR (95% CI)** | **p** | **OR (95% CI)** | **p** |
| Daytime wetting | Self-harm thoughts | 1.94 (0.93, 4.05) | 0.08 | 1.63 (0.76, 3.49)† | 0.2 |
| Bedwetting | Self-harm thoughts | 1.43 (0.55, 3.71) | 0.5 | 1.39 (0.51, 3.74)† | 0.5 |
| Soiling | Self-harm thoughts | 2.35 (1.25, 4.42) | 0.008 | 1.98 (1.03, 3.81)† | 0.04 |
| Urgency | Self-harm thoughts | 1.21 (0.54, 2.71) | 0.6 | 1.05 (0.46, 2.40)† | 0.9 |
| Frequent urination | Self-harm thoughts | 1.52 (0.58, 3.96) | 0.4 | 1.36 (0.50, 3.68)† | 0.5 |
| Low voided volume | Self-harm thoughts | 1.52 (0.74, 3.14) | 0.2 | 1.26 (0.60, 2.65)† | 0.5 |
| Voiding postponement | Self-harm thoughts | 1.42 (0.88, 2.30) | 0.1 | 1.30 (0.79, 2.15)† | 0.3 |
| Nocturia | Self-harm thoughts | 1.90 (1.15, 3.14) | 0.01 | 1.90 (1.13, 3.19)† | 0.01 |
| **Exposure** | **Outcome** | **OR (95% CI)** | **p** | **OR (95% CI)** | **p** |
| Daytime wetting | Fasting | 1.01 (0.39, 2.60) | 0.9 | 0.64 (0.24, 1.71)♦ | 0.4 |
| Bedwetting | Fasting | 1.35 (0.52, 3.52) | 0.5 | 1.10 (0.38, 3.13)♦ | 0.9 |
| Soiling | Fasting | 1.85 (0.96, 3.53) | 0.06 | 1.58 (0.79, 3.13)♦ | 0.2 |
| Urgency | Fasting | 2.13 (1.13, 4.01) | 0.02 | 1.87 (0.94, 3.72)♦ | 0.07 |
| Frequent urination | Fasting | 1.60 (0.70, 3.65) | 0.3 | 1.30 (0.54, 3.13)♦ | 0.6 |
| Low voided volume | Fasting | 1.74 (0.89, 3.42) | 0.1 | 1.48 (0.72, 3.01)♦ | 0.28 |
| Voiding postponement | Fasting | 1.44 (0.91, 2.27) | 0.1 | 1.39 (0.86, 2.26)♦ | 0.2 |
| Nocturia | Fasting | 1.58 (0.92, 2.69) | 0.09 | 1.40 (0.79, 2.49)♦ | 0.2 |
| **Exposure** | **Outcome** | **OR (95% CI)** | **p** | **OR (95% CI)** | **p** |
| Daytime wetting | Purging | 2.16 (0.83, 5.64) | 0.1 | 1.58 (0.58, 4.30)♦ | 0.4 |
| Bedwetting | Purging | 1.58 (0.47, 5.26) | 0.5 | 1.30 (0.36, 4.65)♦ | 0.7 |
| Soiling | Purging | 2.06 (0.91, 4.67) | 0.08 | 1.89 (0.80, 4.45)♦ | 0.1 |
| Urgency | Purging | 1.77 (0.74, 4.24) | 0.2 | 1.55 (0.62, 3.88)♦ | 0.3 |
| Frequent urination | Purging | 1.26 (0.38, 4.17) | 0.7 | 1.01 (0.29, 3.53)♦ | 0.9 |
| Low voided volume | Purging | 2.17 (0.96, 4.93) | 0.06 | 1.98 (0.84, 4.64)♦ | 0.1 |
| Voiding postponement | Purging | 1.36 (0.73, 2.53) | 0.3 | 1.22 (0.64, 2.31)♦ | 0.5 |
| Nocturia | Purging | 2.13 (1.12, 4.08) | 0.02 | 2.01 (1.02, 3.97)♦ | 0.04 |
| **Exposure** | **Outcome** | **OR (95% CI)** | **p** | **OR (95% CI)** | **p** |
| Daytime wetting | Binge-eating | 3.14 (1.62, 6.11) | 0.001 | 2.39 (1.18, 4.83) | 0.01 |
| Bedwetting | Binge-eating | 0.94 (0.33, 2.70) | 0.9 | 0.75 (0.25, 2.25) | 0.6 |
| Soiling | Binge-eating | 1.16 (0.56, 2.38) | 0.7 | 0.96 (0.45, 2.02) | 0.9 |
| Urgency | Binge-eating | 1.20 (0.58, 2.48) | 0.6 | 1.07 (0.50, 2.27) | 0.9 |
| Frequent urination | Binge-eating | 1.20 (0.50, 2.88) | 0.7 | 1.12 (0.45, 2.77) | 0.8 |
| Low voided volume | Binge-eating | 2.39 (1.31, 4.37) | 0.005 | 2.27 (1.21, 4.28) | 0.01 |
| Voiding postponement | Binge-eating | 1.29 (0.82, 2.02) | 0.3 | 1.20 (0.75, 1.91) | 0.4 |
| Nocturia | Binge-eating | 1.52 (0.90, 2.57) | 0.1 | 1.49 (0.86, 2.56) | 0.1 |
| **Exposure** | **Outcome** | **OR (95% CI)** | **p** | **OR (95% CI)** | **p** |
| Daytime wetting | Excessive exercise | 2.07 (1.12, 3.82) | 0.02 | 1.74 (0.91, 3.34) | 0.09 |
| Bedwetting | Excessive exercise | 1.15 (0.53, 2.47) | 0.7 | 1.21 (0.53, 2.78) | 0.6 |
| Soiling | Excessive exercise | 1.25 (0.72, 2.18) | 0.4 | 1.09 (0.60, 1.96) | 0.8 |
| Urgency | Excessive exercise | 0.75 (0.40, 1.43) | 0.4 | 0.77 (0.39, 1.51) | 0.4 |
| Frequent urination | Excessive exercise | 1.15 (0.57, 2.30) | 0.7 | 1.15 (0.55, 2.40) | 0.7 |
| Low voided volume | Excessive exercise | 1.56 (0.91, 2.69) | 0.1 | 1.45 (0.81, 2.58) | 0.2 |
| Voiding postponement | Excessive exercise | 1.42 (1.00, 2.02) | 0.05 | 1.37 (0.94, 1.99) | 0.09 |
| Nocturia | Excessive exercise | 1.39 (0.91, 2.13) | 0.1 | 1.55 (0.99, 2.44) | 0.06 |
| **Exposure** | **Outcome** | **OR (95% CI)** | **p** | **OR (95% CI)** | **p** |
| Daytime wetting | DSM-5 DE | 2.03 (0.93, 4.44) | 0.08 | 1.50 (0.65, 3.47) | 0.3 |
| Bedwetting | DSM-5 DE | 0.85 (0.26, 2.79) | 0.8 | 0.64 (0.18, 2.28) | 0.5 |
| Soiling | DSM-5 DE | 1.87 (0.96, 3.67) | 0.07 | 1.58 (0.77, 3.23) | 0.2 |
| Urgency | DSM-5 DE | 1.11 (0.50, 2.49) | 0.8 | 0.93 (0.40, 2.18) | 0.9 |
| Frequent urination | DSM-5 DE | 2.14 (0.98, 4.71) | 0.06 | 2.09 (0.91, 4.83) | 0.08 |
| Low voided volume | DSM-5 DE | 1.75 (0.87, 3.53) | 0.1 | 1.53 (0.73, 3.24) | 0.3 |
| Voiding postponement | DSM-5 DE | 1.24 (0.76, 2.03) | 0.4 | 1.17 (0.70, 1.98) | 0.5 |
| Nocturia | DSM-5 DE | 1.42 (0.80, 2.52) | 0.2 | 1.31 (0.71, 2.42) | 0.4 |

DSM-5 DE- Disordered eating at Diagnostic and Statistical Manual of Mental Disorders, Fifth Edition frequency (at least once a week). †Analyses performed on n=1494 due to 34 observations being dropped (underweight BMI predicted outcome perfectly). ♦Analyses performed on n=1349 due to 26 observations being dropped (underweight BMI predicted outcome perfectly).

The adjusted model includes the following confounders: sex, socioeconomic indicators, child IQ, developmental level, maternal stressful life events, maternal depression, maternal anxiety, child Body Mass Index and earlier emotional/behaviour problems.
