# Supplementary material for: Continence Problems and Mental Health in Adolescents from a UK Cohort
Source: Eur Urol. Author manuscript; Available in PMC 2024 Dec 9. (PMC7617164; doi:10.1016/j.eururo.2023.05.013)
Supplement: Supplementary Materials [file EMS198154-supplement-Supplementary_Materials.zip › 1-s2.0-S030228382302818X-mmc1.pdf]

Peer Review Overview

Manuscript Title: Continence problems and mental health in adolescents from a UK cohort

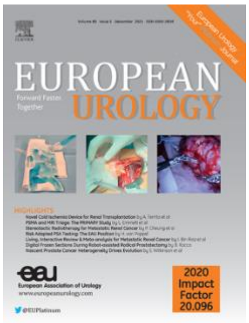

1st Decision letter

Reference: EURUROL-D-23-00016
Title: Continence problems and mental health in adolescents from a UK cohort
Journal: European Urology

Reviewer #1
The authors have performed an interesting study based on the ALSPAC dataset to determine whether there is an association between voiding disorders at age 14 years and mental health disorders at age 18 years.
1.Introduction- line 98, what is the reference for “Most mental health problems begin in adolescence”.
2.Methods- For Continence problems at age 14, it is unclear whether patients were under treatment, whether urotherapy, behavioral management, or pharmacologic therapy. In addition, constipation is a common contributory cause of these problems and this was not assessed in the study, other than asking about soiling (which could result from insufficient wiping).
3.In the Appendix, daytime wetting, nocturnal enuresis, and soiling less than once per week is coded the same as wetting/soiling every day. It seems like it should be stratified.
4.The question did not specifically ask "over the past two weeks".
5.For LUTS, the terms "quite often" and "a lot" seem imprecise.
6.Voiding postponement can be attributed to school bathroom issues, which the authors mention.
7.It is unclear whether patients were under treatment when they answered the questions.
8.The coding for the eating disorders was confusing.
9.Did the dataset screen for autism spectrum disorder or ADHD? Were these patients excluded from the questionnaire study?
10.Results: Table 1—It appears that 3.49% of the 14 yo patients had daytime wetting, 3.04% bedwetting, 4.9% soiling, etc. These are very high percentages.
11.Results: Table 2 appears to come from Table S6 in the Appendix. The authors make statements based on the adjusted data, which is very reasonable.
12.Results: It would be extremely useful to examine the relationship between the severity of daytime wetting, bedwetting and soiling and association with mental health disorders. Unfortunately, unless the ALSPAC database has the raw data and not just the coded data, I do not think that this is possible.
13.It would be a good idea to explore the association between elevated BMI in patients with urinary disorders and subsequent mental health issues.
14.Discussion: The authors should discuss the high prevalence of day and night wetting in the 14-year-old patients. This seems very high compared to other studies.
15.The data presented are interesting, but it would have been much more helpful to understand the mental health answers at age 14 rather than 18 years. It would be inappropriate to attribute mental health disorders in 18-year-old to urinary issues that occurred at age 14.

Reviewer #2
The authors report on the association between incontinence and mental health. The data is derived from a prospective database, with bladder and bowel control data reported at the age of 14, and mental health data reported at 18. I full heartedly agree with the authors' conclusion, stating that providers caring for adolescents (and perhaps younger children, though not directly supported by the presented data) who present with incontinence should appropriately evaluate them for possible mental health issues.
Comments:
1.Throughout the manuscript the authors present the case for incontinence as a cause for mental health

issues. I doubt the data can support that claim. The possibility can be discussed, but cooccurrence rather than causality is what the data suggests.
2.It is not unreasonable that certain mental health conditions may lead to LUTS, (e.g., increased frequency), or that the that the condition simply cooccur.
3.No data is available on treatment for mental health or incontinence.
4.Pharmaceuticals used for mental health have known adverse effects on incontinence and vice versa. Should be discussed in limitations.
5.Both response bias and non-response bias should be discussed in limitations. Participants with urinary issues may be more reluctant to respond to questionnaires regarding incontinence or may be more interested in responding. The same is true for mental health questionnaires.
6.Generalizability is discussed briefly, regarding the socioeconomic conditions. I suggest stating that the data may not reflect the rated of incontinence and mental health status in other parts of the world.

Reviewer #3
According to the Constitution of WHO, health is defined as "a state of complete physical, mental and social well-being and not merely the absence of disease or infirmity." Every concept in this definition is omnidirectional, i.e., sometimes cause, sometimes effect. A holistic approach to health (biopsychosocial approach) is mandatory, and efforts stimulating this approach should be encouraged; just like current manuscript under review. However, the text requires revisions, since some points remains undiscussed, some others overaccentuated, and there seems some confusion that needs to be cleared out. Perhaps, above-mentioned definition of health could serve as a basis and a structure for a better-organized manuscript.
1.In the conclusion section, the authors state that:
"Incontinence/LUTS in young people have long-term consequences for their mental health," hinting that incontinence/LUTS causes mental problems, but they are not certain whether a causal relationship exists. Furthermore, the relationship might be bidirectional, and mental problems might cause LUTS. This aspect deserves some discussion as well.
2.Table 2 includes a vast list of results on "exposure and outcome." If the authors cannot / will not make a biopsychological or philosophical comment on the findings, this large table is not needed.
3.Why did the authors designate a separate area for maternal psychopathology, but no for paternal problems? Did they find anything special to discuss related with this area?
4.The authors used questionnaires evaluating mental health. I would recommend that the paper be reviewed by an expert in this area, if not already done so.

Reviewer #4
There exists ample anecdotal evidence that fundamental problems with urinary continence, a pillar of urologic pathology pursuant to quality of life, embarrass quality of life in general and are frequently observed to have mental health ramifications. This study is an important step in the rigorous analysis of this issue. The authors studied a large cohort of adolescent patients (beginning with over 15,000 subjects) with continence issues at age 14, and the association with the existence of mental health problems 4 years later at age 18. The authors acknowledge this is a purely associative study; next steps would be to seek causal relationships. They used multivariable regression models adjusted for sex, socioeconomic position, developmental level, IQ, stressful life events, maternal psychopathology, body mass index, and earlier emotional/behavioural problems. They found (from Abstract) that daytime wetting and voiding postponement showed the greatest number of associations with mental health problems. All incontinence subtypes/LUTS were associated with statistically significant increased odds of generalised anxiety disorder and/or higher anxiety scores. The study is an important one as it highlights a critical gap in the provision of urological care for these patients since the specialty claims a large part of its rationale is grounded in preserving/improving quality of life. As such, urologists are totally unprepared to manage the mental health consequences of the conditions we treat. This is particularly to be highlighted in the adolescent group which is poised to transition to adult care at a time when transitional services, models, and resources, remain fragmented, mismanaged, and underfunded.
Comments:
1.The lack of any control group. In other words, to what extent would similar adolescents from similar backgrounds without continence issues harbor a proportion of the same mental health conditions noted in the study?
2.The authors should clarify what they mean by adjusting for "earlier" emotional/behavioral problems.
3. Given that the mental health measurements were made at age 18, 4 years after the continence status was determined at age 14, how do the authors know the mental health status is indeed associated with continence

status if the continence status was not validated to still exist unchanged at age 18. What if continence status (as they define) improved in the years between 14 and 18? Are the authors suggesting that the earlier continence issues, even if resolved, might have a lasting detrimental effect on mental health?
4.Overall, an important study given the power of its large numbers, with important conclusions.

1st Author Response Letter
Response to comments from Editors and Reviewers:

Reviewer #1
The authors have performed an interesting study based on the ALSPAC dataset to determine whether there is an association between voiding disorders at age 14 years and mental health disorders at age 18 years.
1.Introduction- line 98, what is the reference for "Most mental health problems begin in adolescence".
Reply: We have added the line:
"Many mental health problems first appear in adolescence".
And have cited the Lancet paper on Adolescence and Mental Health by Sarah-Jayne Blakemore to support this statement (Introduction, line 100).

2.Methods- For Continence problems at age 14, it is unclear whether patients were under treatment, whether urotherapy, behavioral management, or pharmacologic therapy. In addition, constipation is a common contributory cause of these problems and this was not assessed in the study, other than asking about soiling (which could result from insufficient wiping).
Reply: We have added a section to the limitations in the Discussion (lines 253-256) highlighting the "lack of data on treatments for incontinence at age 14 (and possible effects of medications on incontinence), and lack of consideration of constipation as a common contributory cause of continence issues and mental health problems."

3.In the Appendix, daytime wetting, nocturnal enuresis, and soiling less than once per week is coded the same as wetting/soiling every day. It seems like it should be stratified.
Reply: We have added the following section to the limitations in the Discussion (lines 256-263): "This is a community-based sample and, therefore, the number of participants who experienced incontinence at high frequencies (e.g., wetting/soiling every day) is small compared with clinical samples. We therefore examined the presence versus absence of incontinence at any frequency and did not further categorize by frequency of incontinence because this would have resulted in very small group sizes and a lack of precision in our estimates. It is important to note that we found robust associations between incontinence and mental health problems, even when examining incontinence that did not meet the criteria for clinical diagnosis."

4.The question did not specifically ask "over the past two weeks".
Reply: The incontinence questions in the ALSPAC questionnaire are phrased as:
"How often do the following happen to you?"
and the LUTS questions are phrased as:
"Over the last two weeks, how often have you....".
It would have been better if both sets of questions referred to the same time-period. However, it would be reasonable to assume that participants' responses to the incontinence questions reflect their recent experiences.

5.For LUTS, the terms "quite often" and "a lot" seem imprecise.
Reply: We agree that the way in which the ALSPAC questionnaires phrased these questions could be seen as imprecise. However, it is reassuring that the prevalence of continence problems in the ALSPAC cohort not dissimilar to other population-based studies that have examined the prevalence of LUTS in adolescents e.g. J Pediatr Urol. 2019 Apr;15(2): 164.e1-164.e7; Br J Urol 1998;81(Suppl 3):90–3; Br J Urol 1995;76:231–4; J Urol 2012; 188:588–93.

6.Voiding postponement can be attributed to school bathroom issues, which the authors mention.
Reply: Yes, indeed, this is very important. There is evidence for associations between the school toileting environment and LUTS in adolescents (https://pubmed.ncbi.nlm.nih.gov/32679271/). Consistent with this reviewer's statement, we argue in the conclusion that there is a need for access to safe, private, and hygienic toilet facilities to prevent young people from avoiding using school toilets.

7.It is unclear whether patients were under treatment when they answered the questions.
Reply: As mentioned above, data was unavailable on treatments for incontinence at age 14. We have added this as a limitation in the discussion.

8.The coding for the eating disorders was confusing.
Reply: We derived variables for disordered eating (DE) behaviors because the ALSPAC questions did not provide sufficient information to accurately derive research diagnoses of eating disorders. Our method of deriving the DE variables is consistent with other published papers based on the ALSPAC data (e.g., J Affect Disord. 2021 Mar 1; 282:386- 390). ALSPAC questions asked about the compensatory disordered eating behaviours of fasting (not eating for at least a day), excessive exercise (exercise that frequently interfered with daily life) and purging (vomiting or taking laxatives/other medications), in addition to binge-eating (eating an excessive amount with a sense of loss of control). Participants were asked to report the frequency of these behaviours in the past year. We created binary (yes/no) variables based on the responses to these questions. Our primary variable of interest was any disordered eating which was defined as the presence of at least one of the behaviours (fasting, excessive exercise, purging or binge- eating) in the past year. These results are presented in the main manuscript, Table 2. We also used five variables in our secondary analyses. Four of these were the individual disordered eating behaviors, (fasting, excessive exercise, purging or binge-eating) in the past year. The fifth variable, DSM-5 disordered eating, indicates the presence of any of these behaviors (fasting, excessive exercise, purging or binge-eating) at least once a week, which is in line with diagnostic frequency criteria in the DSM-5. These results are presented in Supplementary Tables S8 and S9. We have added the following section to the Method (lines 143-148) to explain this more clearly in the manuscript. The appendix contains full details of how we derived the disordered eating variables (Table S2).

"The Youth Risk Behavior Surveillance System[14] was used to assess disordered eating (DE) behaviours (fasting, purging, binge-eating and excessive exercise). We derived binary variables indicating the presence versus absence of each of these behaviours; a composite variable indicating presence versus absence of any of these behaviours (any DE), and a composite variable for any of the behaviours at the frequency (at least once a week) required for DSM-5 DE diagnosis."

9.Did the dataset screen for autism spectrum disorder or ADHD? Were these patients excluded from the questionnaire study?
Reply: It is important in the clinical care of young people with continence issues to diagnose autism spectrum disorder (ASD) and ADHD because these disorders are associated with incontinence and, according to the International Children's Continence Society "they will interfere with incontinence treatment leading to less favorable outcomes." ALSPAC does identify children with ASD and ADHD, but the proportion of participants meeting diagnostic criteria is very small (e.g., Eur Child Adolesc Psychiatry. 2020 Nov;29(11):1477- 1478.; Dev Med Child Neurol. 2008 Sep;50(9):672-7). We didn't exclude participants with ASD or ADHD because children with these disorders are at higher risk of both incontinence (Neurourol Urodyn. 2022 Jan;41(1):102-114) and mental health problems (J Autism Dev Disord. 2022 Mar;52(3):1077-1091; J Affect Disord. 2021 Nov 1;294:450-458). If we excluded participants with ASD and ADHD, our findings may not generalise to these groups and would hence be of less use clinically where such co-morbidities are not uncommon. Symptoms associated with ASD and ADHD (e.g., developmental delay and behaviour/emotional problems) are quite common in ALSPAC and could be possible common causes of incontinence and mental health problems (i.e. confounders). We therefore adjusted our analysis for these symptoms.

10.Results: Table 1—It appears that 3.49% of the 14 yo patients had daytime wetting, 3.04% bedwetting, 4.9% soiling, etc. These are very high percentages.
Reply: Studies of the prevalence of incontinence in adolescence are rare and definitions of incontinence vary, but the prevalence estimates in ALSPAC are not inconsistent with other studies. For example, the prevalence of daytime wetting was 3.0% in a sample of 15-16-year-old schoolchildren and 1.1% experienced bedwetting (Br J Urol 1998;81(Suppl 3):90–3). A cohort study reported the prevalence of daytime wetting and bedwetting to be 1.1% and 2.7% respectively in adolescence (J Dev Behav Pediatr. 2017 Nov/Dec;38(9):736-742). The prevalence of bedwetting in a large cohort was 3.5% at age 13 (J Paediatr Child Health. 1990 Apr;26(2):75-9). Studies of the prevalence of soiling in adolescence are very rare. The prevalence of soiling was 4.4% in 4-17-year-olds (Arch Dis Child. 2007 Jun;92(6):486-9). In a population-based study, soiling was present in 1.6% of 11-12-year-olds (J Pediatr Gastroenterol Nutr. 2005 Mar;40(3):345-8).

in the discussion (lines 237-241) that soiling was defined by a positive response to the question how often do you "Dirty your pants during the day?" and therefore, it is experienced slightly soiled underwear (rather than an episode of faecal incontinence). We note possible that some young people responded positively to this question if they had only experiences slightly soiled underwear (rather than an episode of faecal incontinence). This could have resulted in some non-differential misclassification of this exposure." We have added a section to explain that the non-differential misclassification could have biased the associations with mental health problems towards the null (lines 240-244).

11.Results: Table 2 appears to come from Table S6 in the Appendix. The authors make statements based on the adjusted data, which is very reasonable.
Reply: Yes, we agree. It is important to base any conclusions on the results following adjustment for the confounders. Table 2 shows the unadjusted and fully adjusted results for the primary analysis and Table S6 shows estimates for all the incremental adjustments.

12.Results: It would be extremely useful to examine the relationship between the severity of daytime wetting, bedwetting and soiling and association with mental health disorders. Unfortunately, unless the ALSPAC database has the raw data and not just the coded data, I do not think that this is possible.
Reply: We agree that this would be useful. However, as mentioned above, categorization according to frequency of incontinence would result in very small group sizes and low statistical power to detect associations.

13.It would be a good idea to explore the association between elevated BMI in patients with urinary disorders and subsequent mental health issues.
Reply: Elevated BMI could be a common cause of both urinary disorders and mental health issues (i.e., a confounder). Therefore, we have adjusted for BMI in our analysis of the association between continence issues and subsequent mental health problems.

14.Discussion: The authors should discuss the high prevalence of day and night wetting in the 14-year-old patients. This seems very high compared to other studies.
Reply: As mentioned above, the prevalence of day and night wetting in ALSPAC is not dissimilar to other population-based studies.

15.The data presented are interesting, but it would have been much more helpful to understand the mental health answers at age 14 rather than 18 years. It would be inappropriate to attribute mental health disorders in 18-year-old to urinary issues that occurred at age 14.
Reply: In clinical care, it is important to know if patients with incontinence experience comorbid mental health problems because these problems can adversely affect treatment outcomes. In a previous study, we examined cross-sectional associations between continence problems and adverse psychosocial outcomes at age 14 (https://www.ncbi.nlm.nih.gov/pmc/articles/PMC5446552/). We have highlighted these cross-sectional findings more clearly in the Introduction. (Lines 96-100): "Cross-sectional associations have been reported between urinary incontinence in adolescence and adverse psychosocial outcomes. For example, compared to young people with no incontinence, those with daytime wetting at 14 years had higher levels of depressive symptoms, peer victimisation, poor self-image, and problems with peer relationships." The aim of our current prospective study is to examine if young people with continence problems at age 14 are at greater risk of developing subsequent mental health problems at 18 years. The motivation behind this aim is to understand if young people with continence problems are vulnerable to developing subsequent mental health problems at an age at which many of them will be transitioning into adult urology services. The prospective design allows us to understand the temporal direction of the association because we are measuring incontinence at time 1 and mental health problems at time 2. We respectfully disagree that it would be inappropriate to attribute mental health disorders in 18-year-olds to urinary issues that occurred at age 14. In the introduction, we review the evidence about the adverse effects of incontinence on young people and argue that they experience a unique myriad of stressors in their daily lives. Stressors such as peer rejection and negative self-beliefs have been found to increase the risk of subsequent mental disorder. As mentioned above, young people with incontinence had higher levels of depressive symptoms at age 14, which has been identified as a risk factor for subsequent major depression and suicidal behaviours (Arch Gen Psychiatry. 2005 Jan;62(1):66-72). The current study takes a developmental perspective to examine the possible long-term mental health

sequalae of experiencing incontinence as a teenager.

Reviewer #2
The authors report on the association between incontinence and mental health. The data is derived from a prospective database, with bladder and bowel control data reported at the age of 14, and mental health data reported at 18. I full heartedly agree with the authors' conclusion, stating that providers caring for adolescents (and perhaps younger children, though not directly supported by the presented data) who present with incontinence should appropriately evaluate them for possible mental health issues.
Reply: It is reassuring that the reviewer strongly agrees with our recommendation to evaluate mental health issues in young people presenting with incontinence.

Comments:
1.Throughout the manuscript the authors present the case for incontinence as a cause for mental health issues. I doubt the data can support that claim. The possibility can be discussed, but cooccurrence rather than causality is what the data suggests.
Reply: We have been careful not to make too strong assertations about causality throughout the manuscript. Prospective studies can determine the direction of association and adjustment for a wide range of confounders (selected using empirical evidence and clinical knowledge) improves casual inference. However, residual confounding is a problem in all observational studies. There are methods that can be used to improve causal inference such as Mendelian randomisation (MR) analysis. MR analysis uses genetic variants that are robustly associated with the exposure as instrumental variables (IVs) to test casual effects. Compared with conventional regression methods, MR analysis is less susceptible to confounding because, at a population level, genetic variants should not be associated with genetic or environmental confounding factors that can distort observational studies. However, MR analysis comes with its own underlying assumptions, strengths and limitations.

2.It is not unreasonable that certain mental health conditions may lead to LUTS, (e.g., increased frequency), or that the that the condition simply cooccur.
Reply: We agree that mental health problems could contribute to LUTS, and we have a programme of research that is currently examining if there are bidirectional relationships between LUTS and mental health problems. It is also possible that mental health problems co-occur with LUTS.

3.No data is available on treatment for mental health or incontinence.
Reply: As mentioned in our response to reviewer 1, data were unavailable on treatments for incontinence at age 14 and we have added this as a limitation in the Discussion.

4.Pharmaceuticals used for mental health have known adverse effects on incontinence and vice versa. Should be discussed in limitations.
Reply: We agree that some medications for mental health problems could have adverse effects on incontinence. There is also evidence that some medications (e.g., for ADHD) reduce incontinence. We have added a comment to the Discussion (lines 253-254) to acknowledge that the lack of data on treatments (including medications) for incontinence is a limitation.

5.Both response bias and non-response bias should be discussed in limitations. Participants with urinary issues may be more reluctant to respond to questionnaires regarding incontinence or may be more interested in responding. The same is true for mental health questionnaires.
Reply: Both scenarios are possible. Reluctance to respond to the questionnaires concerning incontinence and mental health problems would result in selection bias due to missing data. We have addressed possible bias due to missing data using multiple imputation. It is also important to point out that the incontinence and mental health questions were included as parts of much larger ALSPAC questionnaires aimed at collecting data on a wide range of topics.

6.Generalizability is discussed briefly, regarding the socioeconomic conditions. I suggest stating that the data may not reflect the rated of incontinence and mental health status in other parts of the world.
Reply: We have added a sentence to the Discussion (line 274-275): “Research in non-UK samples is also needed to examine if these findings generalize to young people from other countries” and have mentioned this in the Abstract (line 61).

Reviewer #3
According to the Constitution of WHO, health is defined as "a state of complete physical, mental and social well-being and not merely the absence of disease or infirmity." Every concept in this definition is omnidirectional, i.e., sometimes cause, sometimes effect. A holistic approach to health (biopsychosocial approach) is mandatory, and efforts stimulating this approach should be encouraged; just like current manuscript under review. However, the text requires revisions, since some points remains undiscussed, some others overaccentuated, and there seems some confusion that needs to be cleared out. Perhaps, above-mentioned definition of health could serve as a basis and a structure for a better-organized manuscript.
1.In the conclusion section, the authors state that:
"Incontinence/LUTS in young people have long-term consequences for their mental health," hinting that incontinence/LUTS causes mental problems, but they are not certain whether a causal relationship exists. Furthermore, the relationship might be bidirectional, and mental problems might cause LUTS. This aspect deserves some discussion as well.
Reply: We agree that the relationship between incontinence/LUTS and mental health problems could be bidirectional, and we have a programme of research dedicated to examining this. We have already found evidence for prospective relationships between mental health problems and incontinence in children (e.g., Eur Child Adolesc Psychiatry. 2019 Jan; 28 (1): 123-130). We have added a sentence to the discussion to highlight the possibility of bidirectional relationships (line 341-343):
"Research is also needed to examine the possibility of bidirectional casual relationships between mental health problems and incontinence."

2.Table 2 includes a vast list of results on "exposure and outcome." If the authors cannot / will not make a biopsychological or philosophical comment on the findings, this large table is not needed.
Reply: Table 2 includes the main findings from the primary analysis. The table shows the associations (odds ratios and 95% confidence intervals) between continence problems at age 14 (exposures) and mental health problems (outcomes) at age 18. There are 8 exposures (daytime wetting, bedwetting, soiling, urgency, frequent urination, low voided volume, voiding postponement, nocturia) and 7 outcomes (common mental disorder, ICD-10 depression, high depressive symptoms, GAD symptoms, self-harm, self-harm acts, disordered eating). We have reduced the size of the table in the main manuscript because we restricted it to show only the unadjusted and fully adjusted results. The results for incrementally adjusted models are presented in the supplement in Table S6. The Results section describes the findings in this table, and we make detailed comments in the Discussion about the possible biopsychosocial mechanisms that explain the observed associations.

3.Why did the authors designate a separate area for maternal psychopathology, but no for paternal problems? Did they find anything special to discuss related with this area?
Reply: We adjusted the results for maternal psychopathology because there is robust empirical evidence that it is a risk factor for both child incontinence and child mental health problems (e.g., J Am Acad Child Adolesc Psychiatry. 2017 Mar;56(3):250-257; Psychol Rev. 1999 Jul;106(3):458-90.). Adjustment for common causes (i.e., confounders) is important in epidemiological studies to improve causal inference.

4.The authors used questionnaires evaluating mental health. I would recommend that the paper be reviewed by an expert in this area, if not already done so.
Reply: The questionnaires used by ALSPAC to evaluate mental health were selected by the study because they are well-validated and widely used.

Reviewer #4
There exists ample anecdotal evidence that fundamental problems with urinary continence, a pillar of urologic pathology pursuant to quality of life, embarrass quality of life in general and are frequently observed to have mental health ramifications. This study is an important step in the rigorous analysis of this issue. The authors studied a large cohort of adolescent patients (beginning with over 15,000 subjects) with continence issues at age 14, and the association with the existence of mental health problems 4 years later at age 18. The authors acknowledge this is a purely associative study; next steps would be to seek causal relationships.
Reply: Due to the prospective nature of this study and the availability of data on a wide range of important

confounders, our study is higher on the hierarchy of evidence than cross- sectional studies. As mentioned in our response to Reviewer 2 above, residual confounding is a problem in all observational studies, which means that we cannot come to a definitive conclusion about causal effects. Our programme of research is exploring the use of causal inference such as Mendelian randomisation analysis to improve causal inference.

They used multivariable regression models adjusted for sex, socioeconomic position, developmental level, IQ, stressful life events, maternal psychopathology, body mass index, and earlier emotional/behavioural problems. They found (from Abstract) that daytime wetting and voiding postponement showed the greatest number of
associations with mental health problems. All incontinence subtypes/LUTS were associated with statistically significant increased odds of generalised anxiety disorder and/or higher anxiety scores. The study is an important one as it highlights a critical gap in the provision of urological care for these patients since the specialty claims a large part of its rationale is grounded in preserving/improving quality of life. As such, urologists are totally unprepared to manage the mental health consequences of the conditions we treat. This is particularly to be highlighted in the adolescent group which is poised to transition to adult care at a time when transitional services, models, and resources, remain fragmented, mismanaged, and underfunded.
Reply: We are pleased that this reviewer recognises the importance of our findings.

Comments:
1.The lack of any control group. In other words, to what extent would similar adolescents from similar backgrounds without continence issues harbor a proportion of the same mental health conditions noted in the study?
Reply: This is a prospective cohort study (not a case-control study), but our study does include young people with and without continence problems. Prospective cohort studies compare outcomes (i.e., mental health problems) in participants with an exposure (i.e., incontinence/LUTS) to those without the exposure. The study sample at baseline includes 14-year-olds with and without incontinence and follows them up to age 18 years to examine if the odds of mental health problems are greater in young people who had continence problems compared to those without continence problems at age 14. In the appendix we confirm that the continence problems are defined as binary yes/no variables.

2.The authors should clarify what they mean by adjusting for "earlier" emotional/behavioral problems.
Reply: We adjusted for emotional/behaviour problems assessed in the Strengths and Difficulties questionnaire when children were 11 years 8 months old because these are possible common causes of continence problems at age 14 and subsequent mental health problems at age 18 (i.e., they are potential confounders). We have revised the appendix (table S1) to include information on the age at completion of all questionnaires.

3. Given that the mental health measurements were made at age 18, 4 years after the continence status was determined at age 14, how do the authors know the mental health status is indeed associated with continence status if the continence status was not validated to still exist unchanged at age 18. What if continence status (as they define) improved in the years between 14 and 18? Are the authors suggesting that the earlier continence issues, even if resolved, might have a lasting detrimental effect on mental health?
Reply: Our study finds evidence that experiencing continence problems at age 14 is associated with an increased risk of mental health problems at age 18 i.e., the odds of mental health problems at age 18 are greater in participants who experienced continence problems at age 14 compared to those who did not have continence problems. Some participants will experience an improvement in their continence problems between ages 14 and 18. If repeated data were available, a study could examine whether different longitudinal patterns of continence problems are differentially associated with mental health problems. It is plausible, however, that continence issues in teenagers, even if resolved by age 18, could have a lasting detrimental effect on mental health. Our findings are consistent with the conclusion that continence problems in adolescence are associated with an increased risk of subsequent mental health problems. There is evidence that children with a history of incontinence have an increased risk of psychopathology in adolescence, even after the remission of incontinence (J Paediatr Child Health. 1990 Apr;26(2):75-9; J Am Acad Child Adolesc Psychiatry. 2017 Mar;56(3):250-257).

4.Overall, an important study given the power of its large numbers, with important conclusions.

2nd Decision letter

Reference: EURUROL-D-23-00016
Title: Continence problems and mental health in adolescents from a UK cohort
Journal: European Urology

Reviewer #5 (Statistical Editor- Melissa Assel)
Please see our guidelines https://www.europeanurology.com/article/S0302- 2838(18)31002-9/fulltext
Comments:
1.This is important work, and it is critical that the interpretation is one of association not of causality so as to optimize care for these adolescents. The authors make conclusions that suggest the direction of causality is that incontinence/LUTS lead to mental health problems including such statements as:
"Young people with incontinence/LUTS have an increased vulnerability to mental health problems." Or "Our findings have important clinical implications in terms of highlighting the need for provision of psychological support to minimise the risk of mental health problems in young people with incontinence/LUTS." However, the authors haven't established the direction of causality and have no assessments of mental health prior to the establishment of these physiological issues. The authors can say that those with incontinence/LUTS are at an increased risk of mental health issues, but it should be made more explicit throughout the manuscript that we cannot know the direction of causality and it is very well possible that an underlying mental health issue is the cause of physiological issues. I agree that those with these physiological issues should be provided with mental health resources but it could very well be that these adolescents are already suffering so, the mental health resources should assess the patients' current mental health status and not just be geared towards "preventing mental health issues". Please make updates throughout the manuscript and abstract to clarify this for the readers such that the direction of causality is not misrepresented including but not limited to making it clear in the last paragraph of the introduction that their goal is not to establish a causal relationship, update the conclusions and clarify in the interpretation that the direction of causality could reasonable go in either direction and that hasn't been confirmed by this study.
2.Please update the tables to follow guidelines 4.2 and 4.3. Removing redundant rows in Table 1 and reformatting table 2 to remove the repetitive outcome column.
3.Update all tables and results to follow all bullet points in guideline 4.1.
4.Please remove the all sequential or unadjusted models and simply keep the full models per guideline 5.5 (e.g., table 2, supp tables 6- 9).
5.Provide more details about the imputation including what variables were used, how many imputed datasets were generated, and what type of imputation was performed. I don't see any reference to Rubin's rules, typically one performs 10 imputations and uses Rubin's rules to combine results across the multiple imputed datasets.
6.The use of "selection bias" doesn't make sense in this sentence "Attrition could have led to selection bias".
7.Please include a formalized comparison of patient characteristics at age 14 by those who did vs did not respond to the 18-year assessments.
8.Pease update the corresponding limitations section per guideline 6.4

2nd Author Response Letter
Response to comments from Editors and Reviewers:

Reviewer #5 (Statistical Editor- Melissa Assel)
Please see our guidelines https://www.europeanurology.com/article/S0302- 2838(18)31002-9/fulltext
Comments:
1.This is important work, and it is critical that the interpretation is one of association not of causality so as to optimize care for these adolescents. The authors make conclusions that suggest the direction of causality is that incontinence/LUTS lead to mental health problems including such statements as:
"Young people with incontinence/LUTS have an increased vulnerability to mental health problems." Or "Our findings have important clinical implications in terms of highlighting the need for provision of psychological support to minimise the risk of mental health problems in young people with incontinence/LUTS." However, the authors haven't established the direction of causality and have no assessments of mental health prior to the establishment of these physiological issues. The authors can say that those with incontinence/LUTS are at an increased risk of mental health issues, but it should be made more explicit throughout the manuscript that we cannot know the direction of causality and it is very well possible that an underlying mental health

issue is the cause of physiological issues. I agree that those with these physiological issues should be provided with mental health resources, but it could very well be that these adolescents are already suffering so, the mental health resources should assess the patients' current mental health status and not just be geared towards "preventing mental health issues". Please make updates throughout the manuscript and abstract to clarify this for the readers such that the direction of causality is not misrepresented including but not limited to making it clear in the last paragraph of the introduction that their goal is not to establish a causal relationship, update the conclusions and clarify in the interpretation that the direction of causality could reasonable go in either direction and that hasn't been confirmed by this study.
Reply: Thank you for your comments and for the opportunity to further revise our manuscript. We agree that it is critical that our findings are interpreted correctly and have revised the manuscript accordingly. Abstract (page 2 lines 59-60):
Discussion:
"Young people with incontinence/LUTS are at increased risk of mental health problems. Further research is needed to establish the direction of causality."
"Further research is needed to determine the direction of association, since it is also possible that mental health problems could cause incontinence/LUTS." (Page 12, lines 234-236)
"Incontinence/LUTS in young people are associated with an increased risk of mental health problems, and adolescents with daytime wetting and voiding postponement are particularly vulnerable. Our findings have important clinical implications in terms of highlighting the need for provision of psychological support for young people with incontinence/LUTS." (Page 15, lines 306- 309) "Further research is needed to establish whether the associations we have observed are causal and to examine the possibility of bidirectional causal relationships between mental health problems and incontinence." (Page 15, lines 320-322)

2.Please update the tables to follow guidelines 4.2 and 4.3. Removing redundant rows in Table 1 and reformatting table 2 to remove the repetitive outcome column.
Reply: For all the binary variables in Table 1, we provide the prevalence for only one of the two categories (e.g., prevalence of females only) because the prevalence in the other category (males) can be readily derived from the data that have been provided. The three columns in table 1 are necessary because they compare data on the variables in the imputed sample and the samples with complete data. This provides information about the presence of potential selection bias in the samples comprising participants who provided complete data on the exposures, outcomes, and confounders. For example, the prevalence of low parental social class was lower in the samples that were restricted to those with complete data compared with the imputed sample, indicating that the samples with complete data comprised participants who were more socioeconomically advantaged than the imputed sample. Hence, we used multiple imputation to address potential bias caused by missing data and based our findings on the analysis of the imputed dataset.

3.Update all tables and results to follow all bullet points in guideline 4.1.
Reply: We have updated all the tables in the main manuscript and appendix to ensure that we have used appropriate levels of precision and the desired number of significant figures and decimal places.

4.Please remove the all sequential or unadjusted models and simply keep the full models per guideline 5.5 (e.g., table 2, supp tables 6- 9).
Reply: We have removed the sequentially adjusted models as requested. Specifically, we have deleted Table S6 in the Appendix which provided the results for the sequentially adjusted models for the primary analysis. We have also removed the sequentially adjusted models from Table S7, Table S8, and Table S9. We argue that it is necessary to at least show the estimates for the unadjusted and fully adjusted models because differences in the odds ratios could indicate the presence of confounding.

5.Provide more details about the imputation including what variables were used, how many imputed datasets were generated, and what type of imputation was performed. I don't see any reference to Rubin's rules, typically one performs 10 imputations and uses Rubin's rules to combine results across the multiple imputed datasets.
Reply: We have added full details of the imputation model to the Supplementary Materials and provided a reference to Rubin's rules (Appendix S4): (pages and line numbers).
"Appendix S4. Details of the imputation model: Missing data on exposures, outcomes and confounders were imputed using the multivariate imputation by chained equations approach (mi impute chained command in Stata) under the Missing at Random (MAR) assumption. We restricted the sample to those with complete data

on IQ due to a lack of good auxiliary data for IQ, but availability of good auxiliary data for other variables. In addition to variables used in the main analyses, we included auxiliary variables that were likely to be related to the missing data mechanism including incontinence/LUTS (7 and 9 years), depressive symptoms (10 years), depression diagnosis (15 years), emotional problems (7 years), emotional disorder (15 years), anxiety diagnosis (7 years), behaviour/emotional problems (7 and 9 years), self- harm (16 years), suicidal behaviour (11 years), maternal self-harm (during pregnancy), disordered eating (14 and 16 years), and BMI (8, 10, 12.5 years). We also included earlier measures of key indicators of socioeconomic position (material hardship during pregnancy, home ownership and maternal stressful life events at 6 years), maternal mental health (depressive and anxiety symptoms at 2 years) and child developmental level (6 months). We imputed 100 datasets (a decision informed by examining the Monte Carlo errors for the estimated parameters). We repeated analyses across the imputed data sets and combined the estimates using Rubin's rules[1]. Reference:
1. White IR, Royston P, Wood AM: Multiple imputation using chained equations: issues and guidance for practice. Stat Med. 2011; 30:377–399"

6.The use of "selection bias" doesn't make sense in this sentence "Attrition could have led to selection bias".
Reply: We have changed this to: (pages and line numbers).
"Attrition bias due to selective dropout is another potential limitation of this study because the sample with complete data included participants who were more socioeconomically advantaged compared with the original ALSPAC cohort." (Page 12, lines 247-249).

7.Please include a formalized comparison of patient characteristics at age 14 by those who did vs did not respond to the 18-year assessments.
Reply: We have included a formalized comparison of patient characteristics at age 14 by those who did vs did not respond to the 18-year assessments. Specifically, we have added two tables to the appendix: Table S5(i) provides the prevalence of incontinence/LUTS at age 14 by those who did not versus did respond to the 18-year assessments. Table S5(ii) provides the participant characteristics (confounders) by those who did not versus did respond to the 18-year assessments.

8.Pease update the corresponding limitations section per guideline 6.4
Reply: We have checked that our discussion of limitations corresponds to guideline 6.4. We highlight the following sections in the manuscript that refer to bias:
"Attrition bias due to selective dropout is another potential limitation of this study because the sample with complete data included participants who were more socioeconomically advantaged compared with the original ALSPAC cohort. Whilst there is evidence that mental health problems are more common in young people from disadvantaged backgrounds,[21] the evidence concerning the association between incontinence and socioeconomic background is inconsistent.[22] We used multiple imputation to address possible bias due to missing data and compared the results from the analysis of the imputed data and the complete case analysis" (page 12, lines 247-253)
"It is possible that some young people responded positively to this question if they had only experienced slightly soiled underwear (rather than an episode of faecal incontinence), which could have resulted in some non-differential misclassification of this exposure and could, therefore, have biased the associations with mental health problems towards the null." (page 11, lines 224-228)
"There were some notable differences between the analyses based on the imputed compared with the complete data, suggesting that the complete case analysis was biased by missing data and/or was underpowered. For instance, there was evidence in the imputed analysis, but not the complete case analysis, that voiding postponement is associated with CMD, ICD-10 depression, GAD symptoms, and DE." (page 10, lines 189-193)

3rd Decision letter

Reference: EURUROL-D-23-00016
Title: Continence problems and mental health in adolescents from a UK cohort
Journal: European Urology

Reviewer #5
The authors have been very responsive to my prior round of comments. Some further comments:

1.Typically standardized mean differences are presented for the propensity score matched sample as opposed to p-values testing for a difference (https://www.ncbi.nlm.nih.gov/pmc/articles/PMC3472075/). See also "Hypothesis testing and p values are not recommended to check the balance between groups after propensity score matching, since a failure to reject the null hypothesis (i.e., p > 0.05) does not guarantee successful balance of covariates between 2 groups https://www.ncbi.nlm.nih.gov/pmc/articles/PMC4347264/."
2.It would be useful if the authors were to add p-values to the tables testing the difference between responders and non-responders.
3.I don't see a reference to the issue of multiple testing or mention of adjustment to the p-values please make some reference to this in the limitations section per guideline 3.5 and consider whether the conclusions should be updated to address this issue.

3rd Author Response Letter
Response to comments from Editors and Reviewers:

Reviewer #5
The authors have been very responsive to my prior round of comments. Some further comments:
1.Typically standardized mean differences are presented for the propensity score matched sample as opposed to p-values testing for a difference (https://www.ncbi.nlm.nih.gov/pmc/articles/PMC3472075/). See also "Hypothesis testing and p values are not recommended to check the balance between groups after propensity score matching, since a failure to reject the null hypothesis (i.e., p > 0.05) does not guarantee successful balance of covariates between 2 groups https://www.ncbi.nlm.nih.gov/pmc/articles/PMC4347264/."
Reply: The analysis does not include a propensity score matched sample.

2.It would be useful if the authors were to add p-values to the tables testing the difference between responders and non-responders.
Reply: P values have now been added to tables S5(i) and S5(ii) in the supplementary materials.

3.I don't see a reference to the issue of multiple testing or mention of adjustment to the p-values please make some reference to this in the limitations section per guideline 3.5 and consider whether the conclusions should be updated to address this issue.
Reply: We have added this section to the discussion (page 12, lines 246-249):
"Another limitation is the possible increase in Type 1 errors due to multiple testing. However, we have not based our conclusions purely on p value thresholds (e.g. p<0.05) to determine statistical significance, but instead, we consider the effect estimates alongside the strength of evidence indicated by the p values and confidence intervals."
Consistent with many researchers, we do not subscribe to the application of p value thresholds to determine statistical significance (see: Amrhein, V., Greenland, S., & McShane, B. (2019). Scientists rise up against statistical significance. *Nature*, *567*(7748), 305-307. doi.org/10.1038/d41586-019-00857-9). We selected the variables for this study a priori based on previous empirical research on the associations between LUTS/incontinence and mental health and we tested well-defined hypotheses. All the conducted analyses are presented in the manuscript and therefore readers of our manuscript are equipped with the necessary information if they wish to impose a correction.

Accept Letter

Dear Professor Joinson,

We are pleased to inform you that your above-mentioned revised manuscript has been accepted for publication in EUROPEAN UROLOGY. We will forward it to our Publishing Department, which will undergo a desk editing process to ensure the highest quality publication.

If you have not already, you will soon receive a letter detailing the modifications made by the copyeditor and those that need to be addressed when you receive the proofs from the Publishing Department.

We at the Editorial Office of EUROPEAN UROLOGY would like to personally thank you for your interest and support to the Journal, and we do hope that you continue submitting valuable manuscripts to us in the future.

Thank you again for your interest and collaboration with European Urology, The Platinum Journal.

Yours sincerely,

James Catto
Editor-in-Chief

-------- End of Review Comments --------
